# Synchronized crystallization in tin-lead perovskite solar cells

**DOI:** 10.1038/s41467-024-51361-2

**Published:** 2024-08-12

**Authors:** Yao Zhang, Chunyan Li, Haiyan Zhao, Zhongxun Yu, Xiaoan Tang, Jixiang Zhang, Zhenhua Chen, Jianrong Zeng, Peng Zhang, Liyuan Han, Han Chen

**Affiliations:** 1 State Key Laboratory of Metal Matrix Composites, Shanghai Jiao Tong University, Shanghai, China; 2 Innovation Center for Future Materials, Zhangjiang Institute for Advanced Study, Shanghai Jiao Tong University, Shanghai, China; 3 Shanghai Jiao Tong University JA Technology New Energy Materials Joint Research Center, Shanghai, China; 4 Shanghai Synchrotron Radiation Facility, Shanghai Advanced Research Institute, Chinese Academy of Sciences, Shanghai, China; 5 Shanghai Institute of Applied Physics, Chinese Academy of Sciences, Shanghai, China; 6 Joint Research Center for Clean Energy Materials, Shanghai Jiao Tong University, Shanghai, China

**Keywords:** Solar cells, Solar cells

## Abstract

Tin-lead halide perovskites with a bandgap near 1.2 electron-volt hold great promise for thin-film photovoltaics. However, the film quality of solution-processed Sn-Pb perovskites is compromised by the asynchronous crystallization behavior between Sn and Pb components, where the crystallization of Sn-based perovskites tends to occur faster than that of Pb. Here we show that the rapid crystallization of Sn is rooted in its stereochemically active lone pair, which impedes coordination between the metal ion and Lewis base ligands in the perovskite precursor. From this perspective, we introduce a noncovalent binding agent targeting the open metal site of coordinatively unsaturated Sn(II) solvates, thereby synchronizing crystallization kinetics and homogenizing Sn-Pb alloying. The resultant single-junction Sn-Pb perovskite solar cells achieve a certified power conversion efficiency of 24.13 per cent. The encapsulated device retains 90 per cent of the initial efficiency after 795 h of maximum power point operation under simulated one-sun illumination.

## Abstract (web summary)

The film quality of Sn-Pb perovskites is compromised by the asynchronous crystallization. Here, the authors introduce a noncovalent binding agent to target unsaturated Sn(II) solvate, achieving certified efficiency of 24.13% for operationally stable single-junction Sn-Pb perovskite solar cells.

## Introduction

Tin-lead halide perovskites have great photovoltaic potential, either as a single-junction solar cell^1–3^ or as a subcell in all-perovskite tandems^4–7^. Compared to lead perovskites, the incorporation of tin can extend the exploitation of the solar spectrum and reduce toxic lead content. By the principle of detailed balance^8^, mixed Sn-Pb perovskite solar cells (PSCs) possess a higher radiative efficiency limit than the Pb-based analogs. However, the practical efficiency of Sn-Pb PSCs thus far remains below 24%, lagging behind that of the Pb-based devices which have exceeded 26%^9^.

A primary challenge introduced by Sn incorporation, besides the Sn(II) oxidation issue which has been extensively addressed^10,11^, lies in the faster crystallization of Sn-based perovskites compared to the Pb-based component^12,13^. The metal ion-induced nonuniform nucleation and rapid growth result in chemical heterogeneity and optoelectronic defects, substantially compromising film quality and device performance. To reach the full photovoltaic potential of Sn-Pb perovskites, it is crucial to resolve this asynchronous crystallization behavior. Recently, through introducing strongly coordinating ligands, notable progress has been achieved in balancing the crystallization in Sn-Pb perovskites^14–16^. However, the underlying chemistry deriving this disparity between Sn and Pb has yet to be understood.

In this work, we reveal that the sterically active Sn(II) lone pair, which repels Lewis base ligands and destabilizes the high-coordination solvate intermediates, can account for the rapid crystallization of Sn perovskites. In this light, we introduce a noncovalent binding agent that can circumvent the lone pair repulsion and selectively bind to Sn(II) over Pb(II) in the precursor solution. As a result, high-quality Sn-Pb perovskite films with homogenized composition and enhanced optoelectronic properties are produced, enabling a photovoltaic efficiency of 24.5% (certified 24.13%) for single-junction Sn-Pb PSCs, along with improved operational stability.

## Results

### Cause of the asynchronous crystallization

**Fig. 1:**
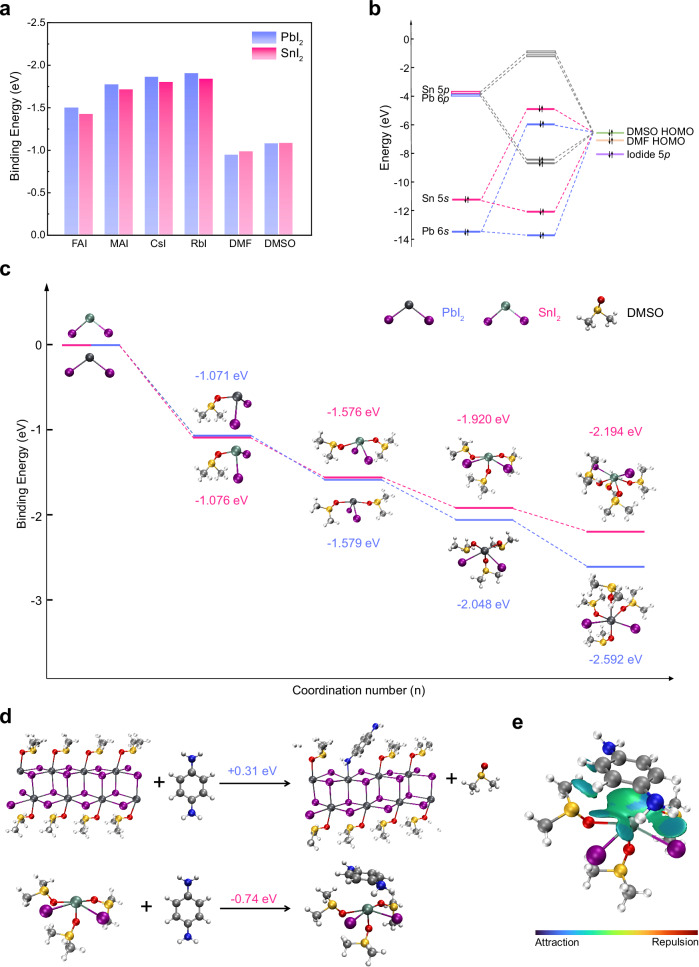
DFT analysis of the BX_2_–ligand interactions. **a** Binding energy of BX_2_ (SnI_2_ and PbI_2_) with the AX iodides and solvent molecules. FAI formamidinium iodide, MAI methylammonium iodide, CsI cesium iodide, RbI rubidium iodide, DMSO dimethyl sulfoxide, DMF dimethylformamide. **b** Frontier orbital interaction diagram of the metal ion-DMSO complex. HOMO highest occupied molecular orbital. **c** Reaction coordinate diagram of BX_2_ binding with different numbers of DMSO ligands. **d** Simulated reactions between PPD and the solvate intermediates (PbI_2_•DMSO and SnI_2_•3DMSO) and the corresponding energy changes. **e** IGMH analysis of the intermolecular noncovalent interactions between PPD and SnI_2_•3DMSO.

Crystallization of halide perovskite (ABX_3_) generally involves the reaction between metal halides (BX_2_) and monovalent halides (AX), where X is (mostly) iodide in photovoltaic devices. According to the Hard and Soft Acids and Bases (HSAB) theory^17^, Sn(II) being a harder acid than Pb(II) implies that Sn(II) has a relatively lower affinity with the soft iodide ion. We calculated the reaction enthalpies between BX_2_ (SnI_2_ and PbI_2_) and common AX iodides through density functional theory (DFT) simulations (Fig. 1a and Supplementary Fig. 1). As indicated by the HSAB theory, compared with PbI_2_, SnI_2_ demonstrates lower binding energies with AX. This suggests that Sn(II) is thermodynamically less favored than Pb(II) for reacting with the AX iodides; the rapid crystallization of Sn perovskites should be more of a kinetic issue.

Since the perovskite thin films are generally solution-processed, an essential factor governing the crystallization kinetics is the solvent-solute interactions, which dictate the structure of the solvate intermediates and hence the activation barrier of the BX_2_-AX reaction. A common practice to retard perovskite crystallization involves the introduction of Lewis base ligands, such as dimethyl sulfoxide (DMSO)^18^, chloride^19^, acetate^20^, formate^21^, thiocyanate^22^, and amino acids^2,23^. These donor ligands can form coordinated covalent bonds with the metal ion, thereby hindering the BX_2_-AX reaction and slowing film growth. However, this Lewis base approach may be relatively less effective for Sn(II) than it is for Pb(II), as discussed below.

Although Sn is above Pb in group 14 on the periodic table, the energy level of Sn 5 *s* orbital is higher than that of Pb 6*s* due to the relativistic contraction of the latter, which also makes the Pb(II) state more stable than Pb(IV). This difference in chemistry between Sn(II) and Pb(II) substantially impacts how the metal ion interacts with the donor ligands. In the frontier orbital perspective, there are mainly two interactions (Fig. 1b): one is between the highest occupied molecular orbital (HOMO) of ligand and the unoccupied metal *np* orbital (gray lines), forming the attractive dative bond; the other is between ligand HOMO and the metal *ns*^2^ lone pair (red/blue lines), which is destabilizing and repulsive due to the occupied anti-bonding orbital. According to the second-order perturbation theory, the strength of the HOMO-*ns*^2^ interaction can be measured based on orbital overlap and energy level separation^24^:

1 $$\Delta E=\frac{{\left|{H}_{{ij}}\right|}^{2}}{{E}_{i}^{0}-{E}_{j}^{0}}$$

where *H*_ij_ is the Hamiltonian matrix element, *E*^0^_i_ and *E*^0^_j_ are the unperturbed energies of the two orbitals. Compared to Pb 6*s*, Sn 5*s* is closer to, or more resonant with, the ligand HOMO, hence the repulsion between Sn(II) lone pair and the ligand is more pronounced^25^. This prominent repulsive orbital interaction is the origin of the narrow bandgap and light holes of Sn perovskites. However, it also impedes the coordination between Sn(II) and Lewis base ligands in the perovskite precursor, limiting the efficacy of the Lewis base approach.

We present the reaction coordinate diagram of BX_2_ coordinated with DMSO (Fig. 1c) and dimethylformamide (DMF, Supplementary Fig. 2), two common solvents employed in perovskite fabrication. When the coordination number is low (small *H*_ij_), the *ns*^2^ lone pair remains largely non-bonding and the stereochemical expression is insignificant. Hence, SnI_2_ exhibits slightly higher binding strength with the solvent molecule than PbI_2_, which can also be understood by the HSAB theory as the oxygen atom (of the solvent) is a hard base. Upon higher coordination numbers, however, the ligand HOMO-*ns*^2^ overlap inevitably increases (large *H*_ij_) and the lone pair stereochemistry becomes significant, making the cohesive energies of the SnI_2_•*n*L (*n* = 2‒4, L = DMSO or DMF) complexes lower than that of PbI_2_•*n*L. Furthermore, a geometric disparity between SnI_2_•4DMSO and PbI_2_•4DMSO highlighting the lone pair activity of Sn(II) is noted; the SnI_2_•4DMSO exhibits a hemi-directed environment while PbI_2_•4DMSO is holo-directed. This distorted coordination of Sn(II) is also observed in some tin chalcogenides (SnS and SnSe)^26^, contrasting with PbS and PbSe which are rock salt structures.

The disparity in the structure of the BX_2_ solvate intermediates has been observed by the community. Experimentally determined Sn(II) solvates in DMF or DMSO are generally isolated complexes and feature coordinatively unsaturated geometry with open Sn sites, including SnI_2_•DMF^27^, SnI_2_•DMSO^27^, SnI_2_•2DMSO^27^, SnI_2_•3DMSO^12^, and Sn(II)•5DMSO^28^. The absence of reports regarding [SnX_6_] suggests the instability of such a hexa-coordinated solvate intermediate in the solution state. As for Pb(II), although the solvate phases can be isolated complexes, bridged chains, or clusters, the local structures are ubiquitously mixed-ligand [PbX_6_] octahedra^29–31^, with X being halide or solvent. For SnI_2_•3DMSO, the SnI_2_ solvate with the highest coordination level, the cohesive energy delivered by the three DMSO ligands was found to be −1.920 eV through DFT, lower than that of the PbI_2_•4DMSO (−2.592 eV). For polyiodide [PbX_6_] in chain or cluster intermediates, the cohesive energy should be comparable to or higher than that of PbI_2_•4DMSO, as the Pb-I bond is stronger than Pb-DMSO dative bond. Therefore, since the BX_2_-AX reaction involves the decomplexation of the solvate intermediates, the crystallization of Sn perovskites should have a lower activation barrier and accordingly a higher rate than that of Pb perovskites.

### Noncovalent binding agent

Given the Sn(II) lone pair disfavoring coordinate covalent bonds, we resort to noncovalent interactions to regulate the crystallization kinetics of Sn(II) perovskites. Noncovalent interactions have been utilized to modulate perovskite crystallization, including hydrogen bond^32^, halogen bond^33^, and anion-π interaction^34^. Amines and nucleophilic aromatic molecules are favorable candidates to bind with the unsaturated Sn(II) solvate through noncovalent forces, such as aniline^35^, piperazine^36^, pyrazine^37^, and aminopyridine^38,39^, which have been proven beneficial in Sn-Pb or neat-Sn perovskites. Here, a simple aromatic amine molecule, p-phenylenediamine (PPD), is employed as the noncovalent binding agent. The resonance effect of PPD, where the nitrogen lone pairs are partially delocalized into the benzene π system, makes the aromatic ring highly nucleophilic and polarizable and thus suitable to establish noncovalent interactions with the Sn(II) solvate, as will be discussed.

We simulated the interactions of PPD with the typical BX_2_ solvate intermediates reported in the literature (Fig. 1d and Supplementary Fig. 3). For PPD to bind with the hexa-coordinated Pb(II) in PbI_2_•DMSO^30^ (Fig. 1d), it would need to substitute a DMSO ligand. However, as the intramolecular resonance reduces the basicity of PPD, such a substitution is thermodynamically unfavored with an energy change of +0.31 eV. For PbI_2_•DMF (Supplementary Fig. 3a), owing to the lower coordinating ability of DMF, the substitution results in an energy change of −0.06 eV. In the PbI_2_•2DMSO chain, where the Pb-Pb distance is shorter than in PbI_2_•DMSO, PPD can form H-bonds with adjacent DMSO ligands, further lowering the energy and yielding an energy change of −0.22 eV. In contrast, for the coordinatingly unsaturated SnI_2_•3DMSO^8^ (Fig. 1d) and SnI_2_•2DMSO^27^ (Supplementary Fig. 3b), PPD can directly occupy the open site without forming a dative bond with the metal ion, yielding binding energies of −0.74 eV and −0.64 eV, respectively.

Ab initio molecular dynamics (AIMD) simulation was conducted to elucidate the dynamic interaction between PPD and the solvate intermediates. The PPD molecule readily engaged and anchored to the SnI_2_•3DMSO intermediate (Supplementary Movie 1), and the resulting complex structure is consistent with the DFT result. Whereas for PbI_2_•DMSO and PbI_2_•2DMSO (Supplementary Movies 2 and 3), PPD primarily interacted with solvent ligands and iodide ions through H-bonds during the 3-µs simulation, with no direct coupling to Pb(II) observed. The DFT and AIMD results indicate that PPD preferentially binds to Sn(II) over Pb(II) in the precursor, both thermodynamically and kinetically.

To gain more insight into the intermolecular interactions between PPD and Sn(II) solvate, symmetry-adapted perturbation theory (SAPT)^40^ was used to decompose the intermolecular interaction energy (Supplementary Table 1), which revealed that electrostatics and dispersion are mainly responsible for the intermolecular attraction, while the charge-transfer contribution is negligible. The Hirshfeld charge of Sn in SnI_2_•3DMSO before and after PPD binding is 0.34*e* and 0.35*e*, respectively, also indicating trivial charge transfer. Furthermore, an independent gradient model based on Hirshfeld partition (IGMH)^41^ was used to visualize the interactions in real space (Fig. 1e). Apart from the hydrogen bonds, a notable attractive force between Sn(II) cation and the aromatic ring is detected, which indicates the cation-π interaction, a noncovalent effect essentially of electrostatic origin and tending to be stronger than hydrogen bond^42^. With these insights, we can discuss the molecular design of the noncovalent binding agent.

To promote intermolecular electrostatics and dispersion, a nucleophilic and polarizable π system is of interest. Besides PPD, molecules with different substituent groups on the aromatic ring were also studied, including benzene, toluene, aniline, phenol, o-phenylenediamine (OPD), and m-phenylenediamine (MPD). The electrostatic potential (ESP) profiles at the van der Waals surface and the local ESP minimum near the ring center were obtained through DFT (Supplementary Fig. 4). Owing to the resonance effect, PPD exhibited the most electron-rich ring center, showing a local ESP minimum of −1.15 eV. The complex structures and binding energies of these agents with SnI_2_•3DMSO were also simulated (Supplementary Fig. 5), where PPD delivered the highest binding energy with Sn(II) solvate. Notably, the binding energy is positively correlated to the local ESP minimum at the ring center (Supplementary Fig. 6), indicating the predominant role of electrostatics in the intermolecular interactions.

### Selective targeting of PPD towards Sn(II)

**Fig. 2:**
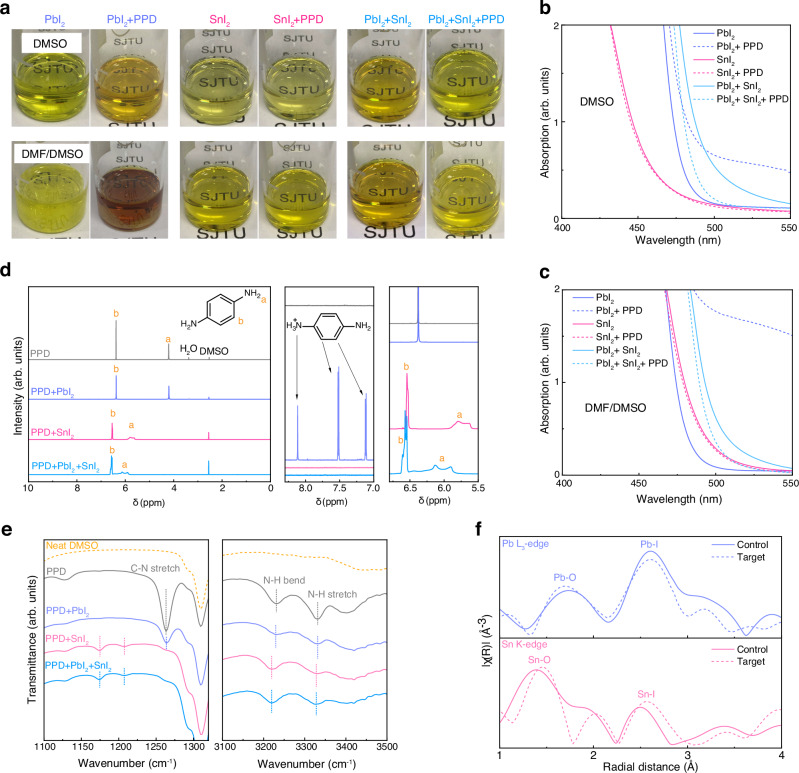
Interactions between PPD and the BX_2_ solvates. **a** Photos of PbI_2_ (1 M), SnI_2_ (1 M), and PbI_2_ + SnI_2_ (0.5 M + 0.5 M) solutions without and with PPD additive (0.08 M). The solutions were stored (N_2_; 20‒30 °C) for 12 h after dissolution. Upper row, DMSO solvent; row below, DMF/DMSO (3:1, v/v) solvent. Note the PbI_2_ needles precipitated in the DMF/DMSO-based PbI_2_ solution, the concentration of which should be lower than 1 M. **b** Absorption spectra of the DMSO-based solutions. **c** Absorption spectra of the DMF/DMSO-based solutions. **d** ^1^H NMR spectra of PPD, PbI_2_ + PPD, SnI_2_ + PPD, and SnI_2_ + PbI_2_ + PPD in DMSO-d_6_, and magnified spectra at the ranges of 5.5‒6.8 ppm and 7.0‒8.3 ppm. **e** FTIR spectra of neat DMSO, and DMSO solutions of PPD, PbI_2_ + PPD, SnI_2_ + PPD, and SnI_2_ + PbI_2_ + PPD. **f** Fourier-transformed radial distribution function of EXAFS spectra of the control and target perovskite precursors.

To explore the interactions between PPD and BX_2_, solutions of BX_2_ without and with PPD were prepared in DMSO and DMF/DMSO (3:1, v/v) solvents. The mixtures were dissolved at 50 °C (Supplementary Fig. 7) and stored for 12 h in an N_2_ atmosphere at ambient temperature for a complete reaction (Fig. 2a–c). In the case of PbI_2_, the incorporation of PPD darkened the solutions and redshifted the absorption spectra. This effect arose from the acid-base reaction between PbI_2_ and protic PPD^43^ (Supplementary Fig. 8). This reaction could be facilitated by the Pb-N dative bond which acidifies the -NH_2_ protons. Conversely, PPD caused a slight blueshift in the SnI_2_ solutions, suggesting that the coupling between PPD and Sn(II) solvates reduced the iodide coordination level of the solvate species.

The mixed SnI_2_ + PbI_2_ solutions exhibited a narrower optical bandgap than both SnI_2_ and PbI_2_ solutions, implying the presence of the bandgap bowing effect in the solution state, through the local Sn-I-Pb bond. With PPD introduced, the SnI_2_ + PbI_2_ solutions also demonstrated blueshifted spectra, indicating that PPD could suppress the formation of the Sn-I-Pb bridge structure. Meanwhile, the lighter color also revealed prevented PPD-PbI_2_ reaction and the selective binding of PPD towards Sn(II). This selectivity was also observed in a mixed Sn(II)/Sn(IV) solution (Supplementary Note 1 and Supplementary Fig. 9). Without a stereochemically active lone pair, Pb(II) and Sn(IV) were fully coordinated by solvent ligands in the solution, and thus exhibited lower affinity with PPD than under-coordinated Sn(II).

Proton nuclear magnetic resonance spectroscopy (^1^H NMR, Fig. 2d) was conducted to investigate the interactions between PPD and BX_2_ in a DMSO solution. When mixing PPD into PbI_2_ solution (DMSO-d_6_), no discernible shift of the protons was observed, suggesting that the majority of PPD had not interacted with the DMSO-coordinated PbI_2_. Meanwhile, three tiny peaks emerged at 7.12, 7.52, and 8.12 ppm and are ascribed to protonated PPD, evidencing the acid-base reaction. In contrast, no signal of protonated PPD is observed in SnI_2_ + PPD, revealing that the acid-base reaction does not occur with SnI_2_. The nucleophilic attack of the -NH_2_ group could be blocked by the Sn(II) lone pair. Additionally, compared to the Pb-I bond, the Sn-I bond has a more covalent character, making the iodine anion harder to ionize.

In the PbI_2_ + SnI_2_ + PPD mixture, the protonated PPD signals were also absent, revealing that SnI_2_ could prevent the reaction between PPD and PbI_2_, as the PPD molecules were captured by SnI_2_ solvates. For the SnI_2_-containing solutions, the PPD protons, especially those of the amine group, exhibited prominent downfield shifts and peak splitting/broadening. The deshielding is caused by the intermolecular H-bonds and the inductive effect of Sn(II) cation, and the peak splitting/broadening should result from the equivalence breaking of the aromatic/amine protons. We also conducted NMR measurements in mixed DMF-d_7_/DMSO-d_6_ and studied the interactions of PPD with FA cation in the perovskite precursors, as discussed in Supplementary Note 2 and Supplementary Figs. 10–12.

Fourier-transform infrared spectroscopy (FTIR, Fig. 2e) measurements were also performed on the DMSO solutions. No discernible vibration shift was observed between the PPD and PPD+PbI_2_ samples, consistent with the NMR result that the majority of PPD agents did not bind with PbI_2_ under the screen of DMSO. In contrast, when PPD was mixed into SnI_2_ and PbI_2_ + SnI_2_ solutions, the vibrations of C-N stretching, N-H bending (overtone), and N-H stretching all shifted to lower wavenumbers. These redshifts were likely caused by the intermolecular coupling between PPD and the Sn(II) solvate, perturbing the intramolecular vibrations.

Extended X-ray absorption fine structure (EXAFS, Fig. 2f) measurements were conducted to detect the local coordination environment of Sn(II) and Pb(II) within the perovskite precursor. The 2 M pristine precursor (Rb_0.04_Cs_0.2_FA_0.76_Pb_0.5_Sn_0.5_I_3_) is denoted as ‘control’ and the precursor doped with PPD (0.08 M) is denoted as ‘target’. Noteworthy, the Pb-O shell exhibited a lower intensity than the Pb-I shell, while the Sn-O shell intensity was higher than the Sn-I shell. This revealed the disparity in the solvate structure between Pb(II) and Sn(II); the Pb(II) solvates tended to form extended structures with polyiodide plumbates [PbX_6_], whereas the Sn(II) solvates were isolated complexes with more solvent ligands in the Sn(II) surrounding.

With PPD incorporated, the Pb-O shell intensity slightly increased, while the Pb-I shell intensity decreased. This suggests increased solvent content and reduced iodide in the [PbX_6_] plumbates, indicating reduced polymeric Pb(II) solvate intermediates in the target precursor. For Sn(II), the Sn-O and Sn-I shells slightly shifted to longer radial distances. This is consistent with the DFT-simulated complex structure, where the average lengths of the Sn-O and Sn-I bonds tend to increase with PPD adsorption (Supplementary Fig. 13). Meanwhile, the ‘Sn-I’ shell of the target precursor exhibited a higher intensity than that of the control. This is attributed to the presence of the PPD molecule, as the distance between Sn(II) and PPD is comparable to the Sn-I bond length.

### Crystallization and film quality

**Fig. 3:**
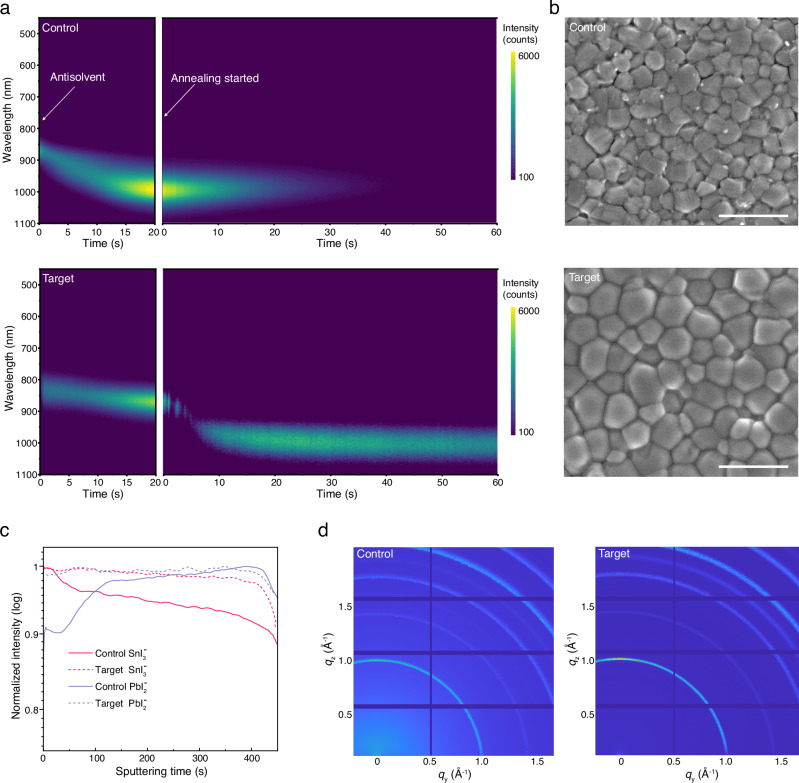
Crystallization process and film quality. **a** In-situ PL monitored during spin-coating (after the anti-solvent was applied) and annealing stages of the perovskite films. **b** Surface SEM images of the control and target perovskite films; the scale bar is 2 µm. **c** Normalized ToF-SIMS depth profiles of the Sn and Pb species in the control and target perovskite films. **d** GIWAXS patterns of the control and target perovskite films.

To investigate the impact of PPD on the crystallization kinetics of the Sn-Pb perovskite, in situ photoluminescence (PL) spectra were collected during spin-coating and annealing (Fig. 3a). The perovskite films were fabricated through the conventional one-step anti-solvent approach. For the control sample, after the anti-solvent (ethyl acetate) treatment, the wet film exhibited a gradually redshifted and broadened emission. The PL peak approached approximately ~1000 nm within 20 s, indicating spontaneous crystallization at room temperature. In contrast, the target wet film at this stage displayed narrower emission spectra and limited evolution of the peak position, demonstrating suppressed crystallization.

During the annealing stage, PL emission of the control film was quenched within 40 sec, resulting from thermally induced defects and subsequent non-radiative recombination. In contrast, the thermal quenching of the target film was significantly slowed down, indicating enhanced crystallinity and heat resistance. Notably, within the initial 5 sec of annealing, the PL intensity of the target film exhibited multiple stages of increase and decrease, accompanied by a prominent redshift. These evolutions demonstrated the occurrence of the crystallization process, and the fluctuations of PL intensity were related to the decomplexation of the intermediate phases^32^.

Scanning electron microscopy (SEM) images of the control and target films are presented in Fig. 3b. Compared with the control, the target Sn-Pb perovskite film exhibits enlarged crystal grains and more compact surface morphology. The enhanced crystallinity is further confirmed by X-ray diffraction (XRD) measurements (Supplementary Fig. 14). The target film showed diffraction peaks with higher intensity and smaller full width at half-maximum than that of the control. No diffraction signal was observed at the low 2θ range, suggesting the absence of low-dimensional perovskite structures. This reveals that the protonated PPD, which induces 2D perovskite^44^, was absent or in a very low content in the perovskite films. PPD has low basicity (conjugate acid p*K*_a_ 6.3) owing to the resonance effect, thus excluding a proton transfer reaction with the FA cation (p*K*_a_ 11.5–13). X-ray photoelectron spectrometry (XPS) measurements (Supplementary Fig. 15) also detected no signal of PPD. Therefore, PPD should be effectively removed from the annealed film.

Time-of-flight secondary ion mass spectrometry (ToF-SIMS) was conducted to detect the composition profile throughout the film depth (Fig. 3c). An evident compositional gradient of Sn and Pb was observed in the control film, with Sn enriched at the upper region of the film (approximately 250 nm depth assuming constant etching speed). This compositional gradient can be understood by the fast crystallization of Sn and the fact that the perovskite film is grown from top to bottom^45^. With PPD applied, the target film exhibited a homogenized distribution of the Sn and Pb along the vertical direction. Meanwhile, the surface Sn/Pb molar ratios derived from the XPS results (Supplementary Fig. 16) were 2.5 and 1.4 for the control and target films, respectively.

To probe this upper region of the perovskite films, synchrotron-based grazing-incidence wide-angle X-ray scattering (GIWAXS) measurements (Fig. 3d) were carried out. With 10 KeV X-rays and a grazing-incidence angle of 0.5°, the penetration depth should be somewhere between 100 and 200 nm^46^. The control film exhibited nearly isotropic diffraction rings, whereas the target sample presented a preferred (100) orientation, indicative of a better-controlled growth. Noteworthy, asymmetric peaks were observed in the control sample (Supplementary Fig. 17), where the long tail indicated the presence of Sn-enriched phases. In contrast, the target film presented symmetric and narrower diffraction peaks, demonstrating enhanced film homogeneity and crystallinity.

Ultraviolet photoelectron spectroscopy (UPS, Supplementary Fig. 18) was conducted to probe the surface electronic structure. The Fermi levels of the control and target films were located at 0.50 eV and 0.59 eV, respectively, above the valence band maximum (VBM), suggesting suppressed p-type doping in the target film. Meanwhile, the target film exhibited a deeper VBM (−5.21 eV) than the control (−5.15 eV). This is consistent with the reduced Sn content at the film surface, as Sn-based perovskite has a more dispersive valence band than the Pb-based analog.

Steady-state PL and time-resolved PL spectra (Supplementary Fig. 19) showed higher emission intensity and elongated carrier lifetimes of the target film compared to the control. Derived from the absorption spectra (Supplementary Fig. 20), the target film featured a smaller Urbach tail (47 meV) than the control (64 meV). Moreover, Kelvin probe force microscopy (KPFM) results (Supplementary Fig. 21) revealed suppressed surface potential fluctuation in the target film, attributed to the improved compositional uniformity and reduced grain boundaries. These results demonstrate enhanced optoelectronic quality of the target film.

### Device performances and stability

**Fig. 4:**
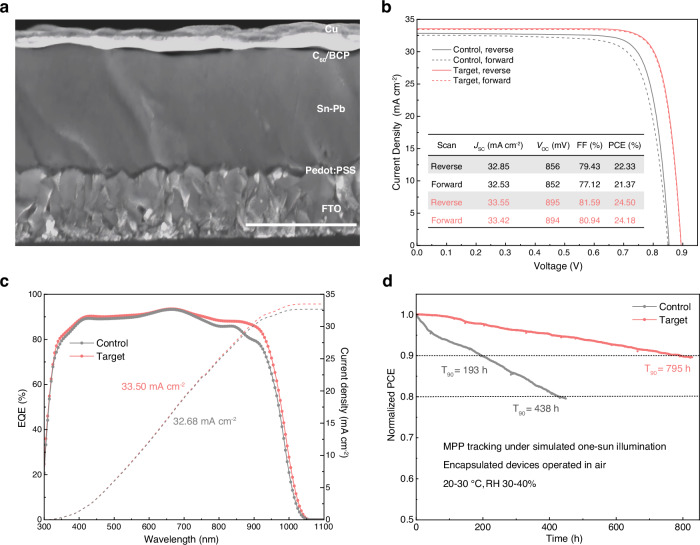
Photovoltaic performances and device stability. **a** Cross-sectional SEM image of a single-junction Sn-Pb PSC; the scale bar is 1 µm. **b** *J*–*V* curves of the control and target devices, and the corresponding photovoltaic parameters in reverse and forward scan. **c** External quantum efficiency spectra and integrated *J*_SC_ of the control and target devices. **d** Continuous maximum power point tracking of the encapsulated devices.

We fabricated single-junction Sn-Pb PSCs with the architecture fluorine-doped tin oxide (FTO)/Poly(3,4-ethylenedioxythiophene) polystyrene sulfonate (PEDOT:PSS)/Sn-Pb perovskite/C_60_/bathocuproine (BCP)/Cu (Fig. 4a). The highest efficiency achieved by the target device (0.0916 cm^2^) was 24.18% in forward scan and 24.50% in reverse scan (Fig. 4b), whereas the control device showed a power conversion efficiency (PCE) of 21.37% in forward scan and 22.33% in reverse scan. The target device also demonstrated a stabilized output efficiency of 24.4% (Supplementary Fig. 22). The champion device was measured in an accredited laboratory (Test and Calibration Centre of the New Energy Device and Module, Chinese Academy of Sciences). The certified PCE was 24.13% in the reverse scan and 23.59% in the forward scan (Supplementary Fig. 23). The effect of the PPD concentration was investigated (Supplementary Fig. 24), and the highest average PCE was achieved with 8 mol% (with respect to SnI_2_) PPD incorporated. Several other agents were also studied (Supplementary Fig. 25), and aniline was also found to deliver a prominent impact on device performance. Moreover, PPD was extended to neat Sn-based perovskites and demonstrated a substantial improvement in the device efficiency (Supplementary Fig. 26).

External quantum efficiency (EQE) spectra (Fig. 4c) revealed that the *J*_SC_ increase mainly originated from EQE enhancement at the infrared range (750–950 nm). This is attributed to the improved film quality at the upper region (rear part) of the perovskite absorber. Through the inflection point method^47^, bandgaps of the control and target films were determined as 1.259 eV and 1.253 eV, respectively (Supplementary Fig. 27). The sharpened absorption edge and narrowed bandgap revealed reduced band tail states within the target film.

We monitored the efficiency evolution of 16 unencapsulated target cells during a six-month storage in N_2_ atmosphere under ambient temperature (following the ISOS-D-1I protocol^48^; Supplementary Fig. 28). The average PCE of the freshly fabricated devices reached the highest point after six days of storage. The devices retained > 97% of the highest average PCE after six months of storage. Maximum power point tracking (MPPT, Fig. 4d) following the ISOS-L-1I protocol was conducted with the encapsulated devices under simulated one-sun illumination (Xenon lamp simulator, Supplementary Fig. 29) in ambient air. The PCE of the control device declined to 80% after 438 h of aging, whereas the target device retained 90% of its initial PCE after 795 h. This result stands as a remarkable MPPT performance among single-junction Sn-Pb PSCs recently reported (Supplementary Table 2). The stability enhancement is attributed to the suppressed surface Sn-enrichment and enhanced crystallinity, which could suppress oxidative degradation and moisture intrusion at grain boundaries^49^ (Supplementary Fig. 30).

## Methods

### Computational methods

DFT and AIMD simulations of isolated complexes were conducted with the ORCA5.0.3 package^50^. Geometry optimizations were performed at the M06-2X^51^/Def2-TZVP^52,53^ level of theory with D3zero^54^ dispersion correction. The single point energies were obtained at the PWPB95^55^-D4^56^/Def2-TZVPP^57^ level of theory. AIMD was performed at the ωB97X-D3^58^/def2-SVP^52^ level of theory. The temperature was maintained at 300 K by the Nosé-Hoover chain^59^ thermostat and the time step was set to 1.0 fs. Simulations of the crystalline PbI_2_ solvates (periodic structures) were conducted by CP2K9.1^60^. Geometry optimization and AIMD were performed using PBE^61^ functional with DFT-D3^54^ dispersion correction. The Brillouin zone is sampled at the gamma point and the plane wave cutoff energy is 500 Ry. The temperature was maintained at 300 K by the CSVR^62^ thermostat and the time step was set to 1.0 fs. SAPT analysis at the SAPT2 level was carried out by PSI4^63^ code. The Multiwfn^64^ code was used for the ESP and IGMH analysis. Visualization was conducted by Visual Molecular Dynamics (VMD)^65^.

### Materials

Dimethylformamide (DMF, anhydrous 99.8%), dimethyl sulfoxide (DMSO, >99.9%), methanol (MeOH, anhydrous 99.8%), ethylene glycol (EG, anhydrous 99.8%), ethyl acetate (EA, anhydrous 99.8%), isopropyl alcohol (IPA, anhydrous 99.5%), cesium carbonate (99.9%), cesium iodide (99.999%, anhydrous), ethylenediammonium diiodide (EDAI_2_, 99%), rubidium iodide (RbI, 99.9%), SnF_2_ (99%), aniline (>=99.5%), and p-phenylenediamine (PPD, >=99%) are purchased from Sigma-Aldrich. Toluene (99.5%) is obtained from Sinopharm. PEDOT:PSS (Clevious P VP AI 4083) is obtained from Heraeus. SnI_2_ (99.99%) is obtained from Advanced Election Technology. Lead iodide (PbI_2_, 99.99%), phenol (99.5%), and ammonium thiocyanate (NH_4_SCN, 98%) are purchased from TCI. Formamidinium iodide (FAI) is purchased from Greatcell Solar Materials. C_60_, indene-C_60_-bisadduct (ICBA), phenylethylammonium bromide (PEABr), and phenethylammonium chloride (PEACl) are purchased from Xi’an Yuri Solar. Bathocuproine (BCP) is obtained from Wako Chemical. The commercial SnI_2_ powder was purified via a reported toluene washing method^66^. Other materials were used as received.

### Precursors preparation

Sn-Pb perovskite (Rb_0.04_Cs_0.2_FA_0.76_Pb_0.5_Sn_0.5_I_3_): The 2 M precursor was prepared by mixing RbI (0.08 mmol), CsI (0.4 mmol), FAI (1.52 mmol), PbI_2_ (1 mmol), SnI_2_ (1 mmol), SnF_2_ (0.1 mmol), NH_4_SCN (0.045 mmol), and PEACl (0.008 mmol) into 750 µL of DMF and 250 µL of DMSO. For the target sample, 8 mol% PPD (0.08 mmol) regarding SnI_2_ was added into the precursor. The precursors were dissolved at 50 °C overnight and filtered through a 0.22-μm PTFE filter before use.

Sn perovskite (PEA_0.15_FA_0.85_SnI_2.85_Br_0.15_): The 0.8 M precursor was prepared by dissolving PEABr (0.12 mmol), FAI (0.68 mmol), SnI_2_ (0.8 mmol), SnF_2_ (0.06 mmol), NH_4_SCN (0.04 mmol), and EDAI_2_ (0.008 mmol) in 800 µL of DMF and 200 µL of DMSO. For the target sample, PPD (0.064 mmol) was added to the precursor. The precursor was dissolved at 50 °C overnight and filtered through a 0.22-μm PTFE filter before use.

PEDOT:PSS dispersion: The commercial PEDOT:PSS was mixed with cesium carbonate at the ratio of 1 mL: 2 mg, and the mixture was filtered through a 0.45-μm PVDF filter.

Surface passivating solution: The EDAI_2_ solution was prepared by mixing EDAI_2_ (3 mg) with IPA (2 mL) and toluene (2 mL), heated at 50 °C overnight, and filtered through a 0.22-μm PTFE filter before use.

### Devices fabrication

Sn-Pb PSCs: Pre-patterned FTO substrates (AGC, 6‒8 Ω) were cleaned using deionized water, acetone, and IPA under ultrasonication. The dried substrates were treated with Ar/O_2_ (1:1, v/v) plasma for 5 mins. The PEDOT:PSS dispersion was spin-coated onto the FTO substrates at 6000 rpm for 40 s. After air drying for 20 min, a mixed solvent of MeOH/EG (50:1, v/v) was applied on the deposited PEDOT:PSS and spin-coated at 6000 rpm for 30 s, followed by annealing at 160 °C for 20 min in air. The substrates were immediately transferred to the N_2_-filled glovebox after annealing. The perovskite precursor was spin-coated with a two-step procedure. The first step was at 1000 rpm for 10 s with an acceleration of 200 rpm·s^−1^; the second step was at 4000 rpm for 50 s with an acceleration of 1000 rpm·s^−1^. Ethyl acetate (350 μL) was dropped 20 s before the end of the spin, and then the substrates were annealed at 100 °C for 10 min. The EDAI_2_ solution was applied onto the cooled perovskite films and spin-coated at 4000 rpm for 25 s, followed by annealing at 100 °C for 5 min. C_60_ (20 nm), BCP (7 nm), and Cu (100 nm) in sequential order were thermally evaporated in a vacuum chamber ( < 3 × 10^−4 ^Pa).

Sn PSCs: FTO substrates cleaning, plasma treatment, and the PEDOT:PSS layer deposition followed the same procedures as the Sn-Pb PSCs. The Sn perovskite precursor was spin-coated with a two-step procedure. The first step was at 1000 rpm for 10 s with an acceleration of 200 rpm·s^−1^, and the second step was at 5000 rpm for 32 s with an acceleration of 1000 rpm·s^−1^. Toluene (600 μL) was dropped 10 s before the end of the spin, and then the substrates were annealed at 80 °C for 10 min. ICBA (5 mg mL^−1^ in chlorobenzene) was spin-coated onto the perovskite film at 5000 rpm for 40 s and annealed at 70 °C for 5 min. C_60_ (18 nm), BCP (7 nm), and Cu (100 nm) in sequential order were thermally evaporated in a vacuum chamber ( < 3 × 10^−4 ^Pa).

### Solar cell characterizations

*J–V* curves of the perovskite solar cells were measured with a Keithley 2400 source meter under simulated one-sun illumination from a dual light source simulator (WXS-90S-L2, Wacom). The light intensity was calibrated by a mono-Si reference cell (91150-KG5, calibrated by Newport). The spectral mismatch factor was controlled within 0.99‒1 at the wavelength range of 350‒1100 nm. The PCE measurements were conducted in dry air (RH 10‒20%, environment temperature 20‒30 °C), and no preconditioning was performed. *J–V* curves were measured in reverse scan (1.0 V to −0.1 V) and forward scan (−0.1 V to 1.0 V) under a constant scan speed of 100 mV·s^−1^ with voltage steps of 10 mV and a delay time of 100 ms. A metal aperture (0.0916 cm^2^) was used to define the active area of the devices. The stable power output efficiency was recorded by monitoring the output efficiency with the biased voltage set at the maximum power point. External quantum efficiency was obtained using monochromatic incident light of 1 × 10^16^ photons cm^−2^ in direct current mode (CEP-2000BX, Bunko-Keiki). Anti-reflecting coating layers (Mosmite, Mitsubishi) were applied for the champion cells during PCE and EQE measurements.

### Encapsulation and stability test

For device encapsulation, a polytetrafluoroethylene (PTFE) layer (~10 µm, measured by a profilometer) was deposited on the device (leaving the electrode contact uncovered) through thermal evaporation (Supplementary Fig. 31). Then glass-to-glass sealing using a caved glass cover and epoxy sealing was conducted to complete the encapsulation. For the shelf stability measurement, the unencapsulated devices were stored in the N_2_-filled glovebox (H_2_O, <0.01 ppm; O_2_, <0.01 ppm) in the dark. For maximum power point tracking, the encapsulated device was operated at the *V*_MPP_ voltage (0.76 V) in ambient air (RH 30‒40%, temperature 20‒30 °C) under simulated one-sun illumination (Xenon lamp; SS-X100R, Enlitech).

### Other characterizations

GIWAXS measurements were performed at the BL17B beamline of the Shanghai Synchrotron Radiation Facility (SSRF) using 10 KeV X-rays. The grazing-incidence angle was 0.5° and the exposure time was 20 s. The EXAFS spectra were collected at the BL13SSW beamline in SSRF. The measurements were conducted under transmission mode with a germanium solid-state detector. The X-ray energy was calibrated by the absorption edge of pure Sn/Pb foil. Fourier-transformed radial distribution functions of *k*^2^-weighted EXAFS spectra were obtained in the *k* range between 3 and 12.5 Å^−1^ through the ATHENA^67^ code.

The NMR spectra were measured on an Avance III 400 MHz NMR spectrometer, using DMSO-d_6_ as the solvent. The FTIR measurements were conducted using the attenuated total reflectance (ATR) method on a Nicolet 6700 spectrophotometer. For sampling, a small drop of the solution was placed onto the ATR crystal. The crystal was wiped clean using DMSO and ethanol after each measurement. In situ PL measurement was conducted with a homebuilt setup in glovebox. A diode laser (405 nm, 50 mW) was introduced onto the sample through an optical fiber, and the emission spectra were collected by a fiber-coupled with a USB 2000+ spectrometer (450‒1100 nm, Ocean Optics), calibrated by the local vendor. A 450-nm optical filter is applied between the sample and the fiber detector. ToF-SIMS experiments were performed using a ToF-SIMS 5-100 (ION TOF) instrument. The sputter ion was 20 keV Cs ion and a 400 × 400 µm^2^ sputter crater was used. A 30 keV Bi^3+^ ion beam with an ion current of 0.45 pA was applied over a 100 × 100 µm^2^ area at the center of the sputter crater.

Absorption spectra were recorded by a UV–Vis–NIR spectrometer (PerkinElmer Lambda 750 S). The solutions were stored in sealed quartz vials during absorption measurement. The SEM images of perovskite films were obtained using a JSM‐7800F instrument. The X-ray diffraction (XRD) was conducted using an X-ray diffractometer (Ultima IV) with Cu Kα radiation at a scan speed of 2° min^−1^. X-ray photoelectron spectroscopy (XPS) measurements were performed on a Kratos AXIS Ultra DLD spectrometer using an Al Ka X-ray source. Au layer (5 Å) was deposited on the sample through thermal evaporation and the spectra were calibrated with Au 4*f*_7/2_ binding energy (84.0 eV). Ultraviolet photoelectron spectroscopy (UPS) spectra were measured with a He Iα photon source. The binding energy scale was calibrated using a clean gold film. PL spectra and time-resolved PL spectra were recorded by a fluorescence spectrometer (FLS1000). AFM morphology and KPFM measurements were conducted on the FastScan Bio (Bruker).

### Reporting summary

Further information on research design is available in the Nature Portfolio Reporting Summary linked to this article.

## Supplementary information

Supplementary Information

Peer Review File

Description of Additional Supplementary Files

Supplementary Movie 1

Supplementary Movie 2

Supplementary Movie 3

Reporting Summary

## Data Availability

The source data underlying Fig. 4b–d, Supplementary Fig. 24, and Supplementary Fig. 28 have been deposited in the Figshare repository (10.6084/m9.figshare.26150392). Atomic coordinates of the DFT-optimized structures and AIMD trajectories are available from Materials Cloud (10.24435/materialscloud:kv-g1). All other data of this study are available from the corresponding author. Source data are provided in this paper.
