# Author Correction: Synchronized crystallization in tin-lead perovskite solar cells

**DOI:** 10.1038/s41467-024-52796-3

**Published:** 2024-09-25

**Authors:** Yao Zhang, Chunyan Li, Haiyan Zhao, Zhongxun Yu, Xiaoan Tang, Jixiang Zhang, Zhenhua Chen, Jianrong Zeng, Peng Zhang, Liyuan Han, Han Chen

**Affiliations:** 1 State Key Laboratory of Metal Matrix Composites, Shanghai Jiao Tong University, Shanghai, China; 2 Innovation Center for Future Materials, Zhangjiang Institute for Advanced Study, Shanghai Jiao Tong University, Shanghai, China; 3 Shanghai Jiao Tong University JA Technology New Energy Materials Joint Research Center, Shanghai, China; 4 Shanghai Synchrotron Radiation Facility, Shanghai Advanced Research Institute, Chinese Academy of Sciences, Shanghai, China; 5 Shanghai Institute of Applied Physics, Chinese Academy of Sciences, Shanghai, China; 6 Joint Research Center for Clean Energy Materials, Shanghai Jiao Tong University, Shanghai, China

**Keywords:** Solar cells, Solar cells

Correction to: *Nature Communications* 10.1038/s41467-024-51361-2, published online 12 August 2024

The original version of this article contained an error in Fig. 1, in which the labels at the bottom left corner (within the −10 to −14 eV range) in the Fig. 1b plot were incorrect. The correct text should be “Sn 5*s*” and “Pb 6*s*” instead of “Sn 5*p*” and “Pb 6*p*”, respectively.

The correct version of Fig. 1 is:

**Figure:**
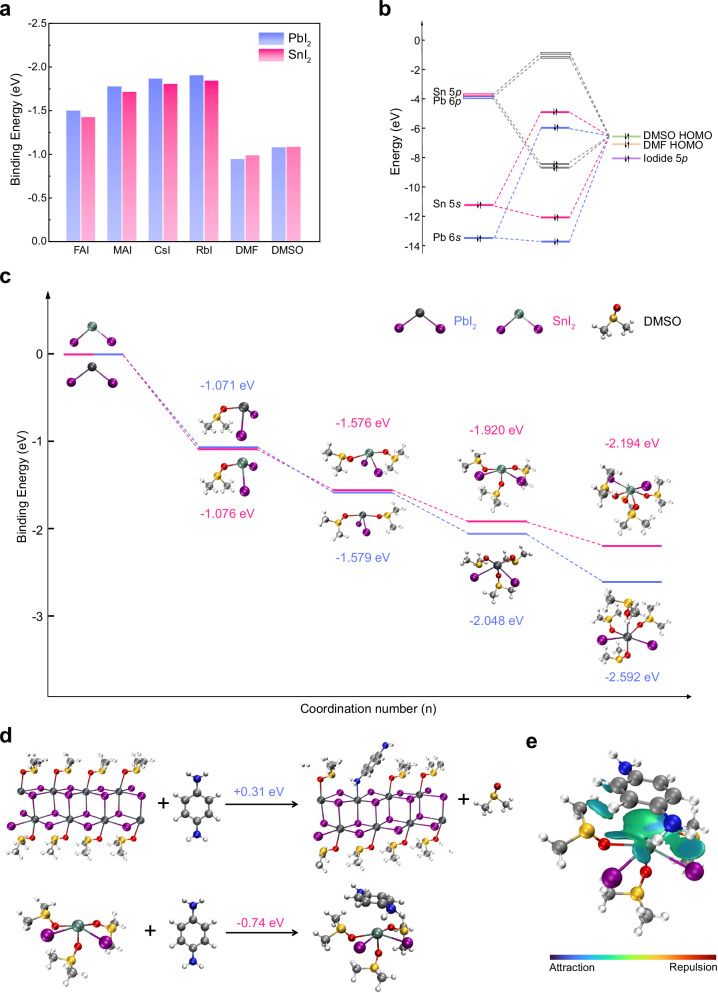

which replaces the previous incorrect version:

**Figure:**
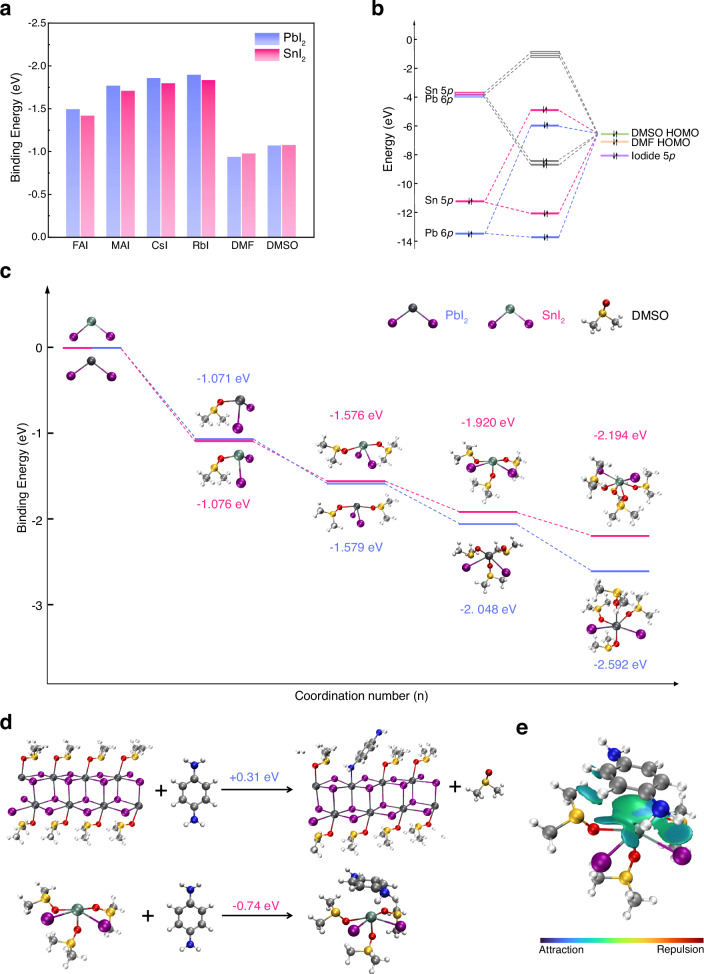

This has been corrected in both the PDF and HTML versions of the Article.

