# Supplementary material for: Synchronized crystallization in tin-lead perovskite solar cells
Source: Nat Commun. 2024 Aug 12;15:6887. doi: 10.1038/s41467-024-51361-2 (PMC11319464; doi:10.1038/s41467-024-51361-2)
Supplement: Supplementary file 1 — Supplementary Information [file 41467_2024_51361_MOESM1_ESM.pdf]

Supplementary Information for:

Synchronized crystallization in tin-lead perovskite solar cells

Yao Zhang[1,2,†], Chunyan Li[1,2,†], Haiyan Zhao[1,2], Zhongxun Yu[1,2,3], Xiaoan Tang[1,2], Jixiang Zhang[1,2,3], Zhenhua, Chen[4], Jianrong, Zeng[4,5], Peng Zhang[1,6], Liyuan Han[1], Han Chen[1,2,6*]

[1]State Key Laboratory of Metal Matrix Composites, Shanghai Jiao Tong University, Shanghai, China.

[2]Innovation Center for Future Materials, Zhangjiang Institute for Advanced Study, Shanghai Jiao Tong University, Shanghai, China.

[3]Shanghai Jiao Tong University JA Technology New Energy Materials Joint Research Center, Shanghai, China.

[4]Shanghai Synchrotron Radiation Facility, Shanghai Advanced Research Institute, Chinese Academy of Sciences, Shanghai, China.

[5]Shanghai Institute of Applied Physics, Chinese Academy of Sciences, Shanghai, China.

[6]Joint Research Center for Clean Energy Materials, Shanghai Jiao Tong University, Shanghai, China.

[†]These authors contributed equally: Yao Zhang, Chunyan Li

*Correspondence: chen.han@sjtu.edu.cn (H.C.)

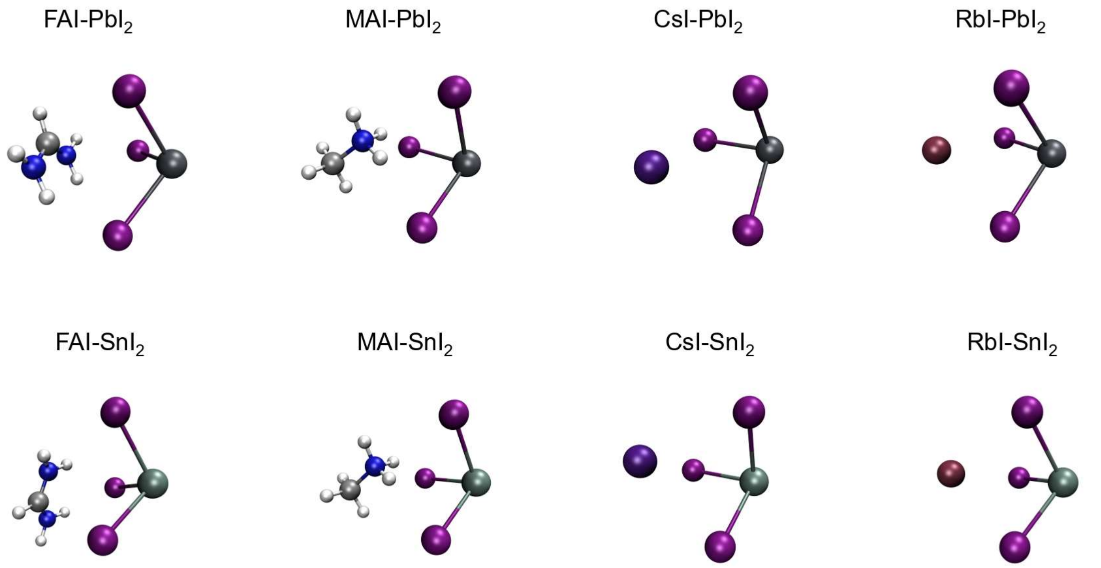

Supplementary Fig. 1. Optimized complex structures of BX_2 (SnI_2 and PbI_2) binding with AX iodides (FAI, MAI, CsI, and RbI).

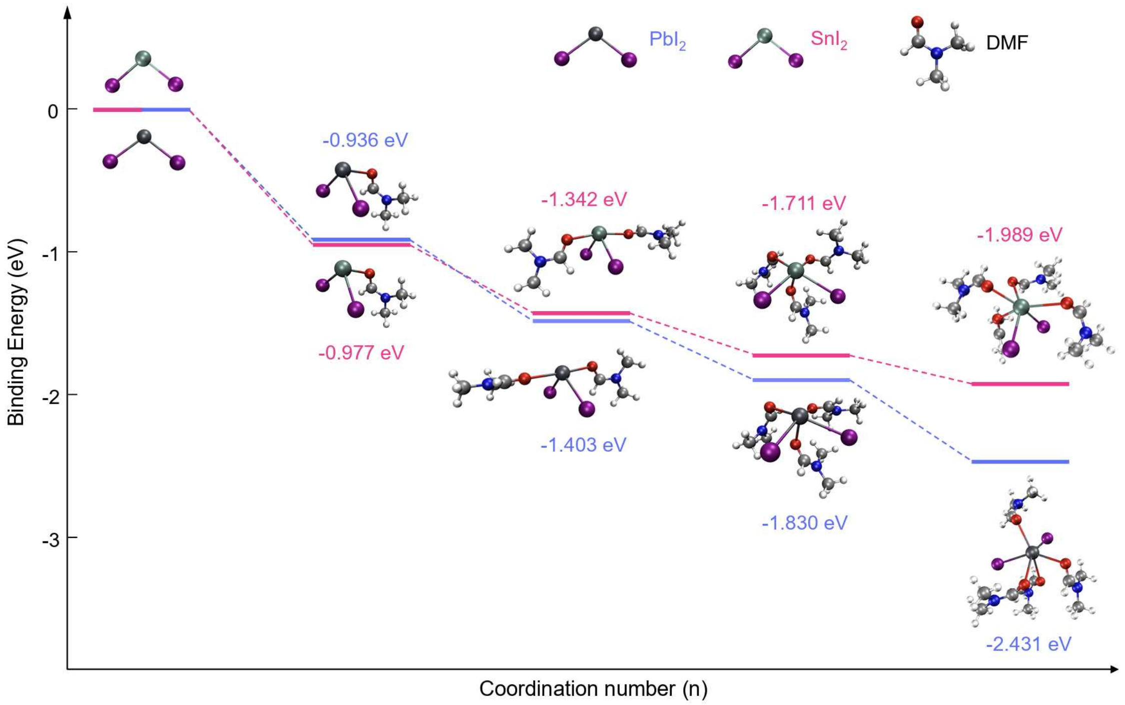

Supplementary Fig. 2. Reaction coordinate diagram of SnI_2 and PbI_2 binding with DMF molecules.

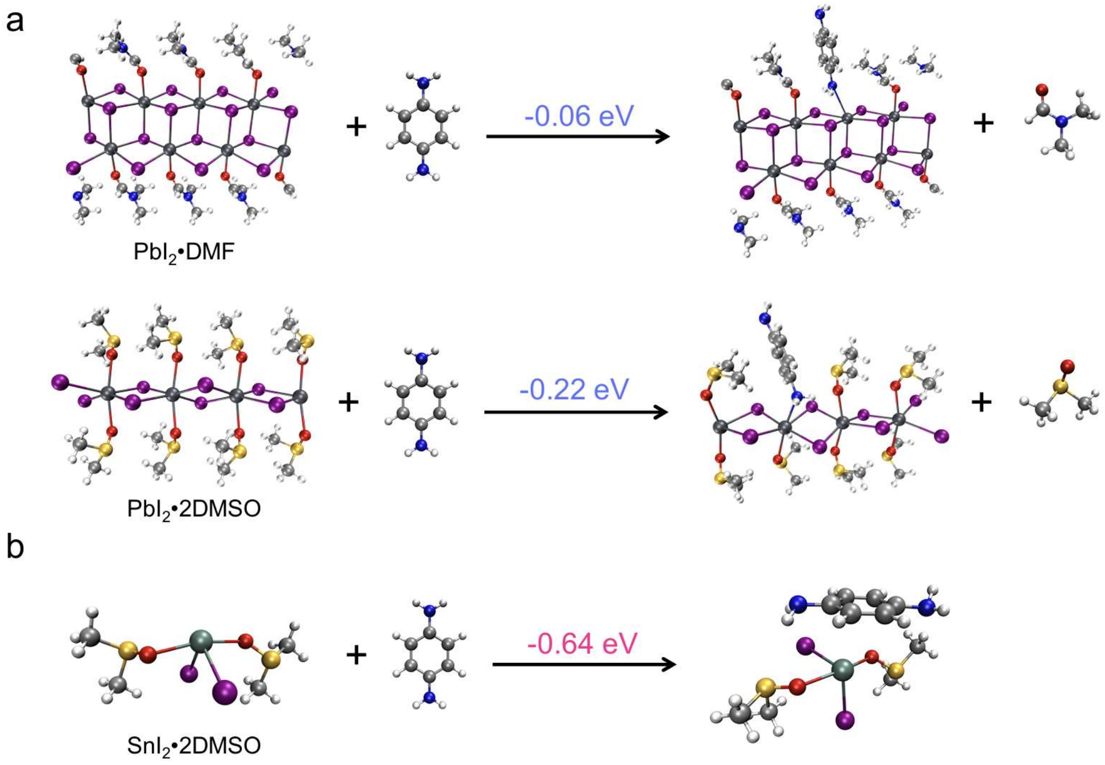

Supplementary Fig. 3. Reactions of PPD with the solvate intermediates. a, PbI_2•DMSO and PbI_2•2DMSO; **b**, SnI_2•2DMSO.

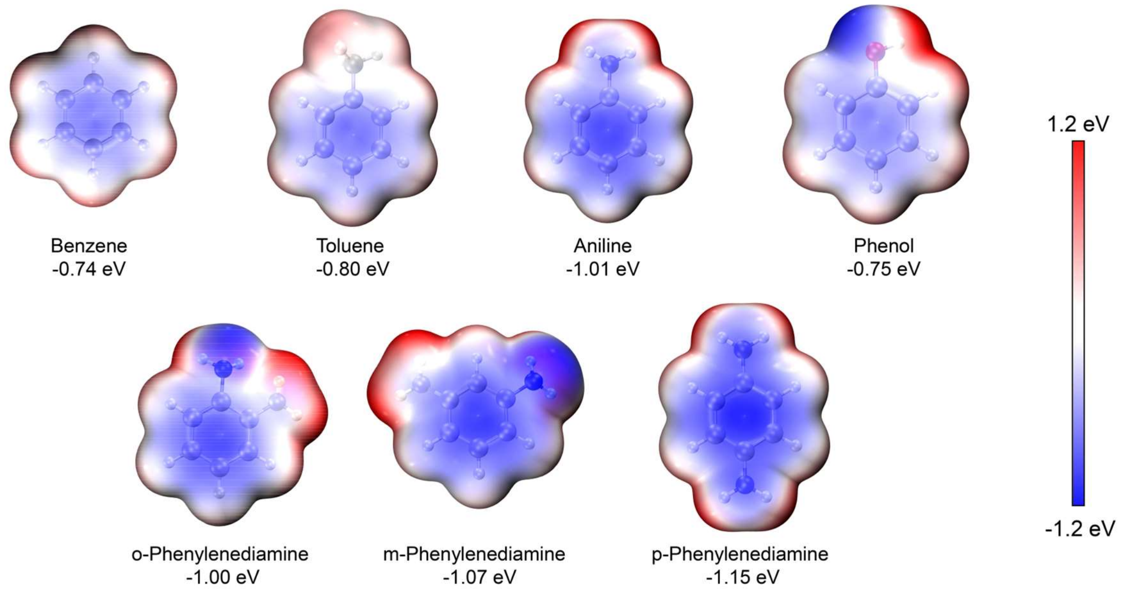

Supplementary Fig. 4. Electrostatic potential (ESP) profiles on the van der Waals surface of the molecules. The values attached below indicate the local ESP minimum near the center of the benzene rings.

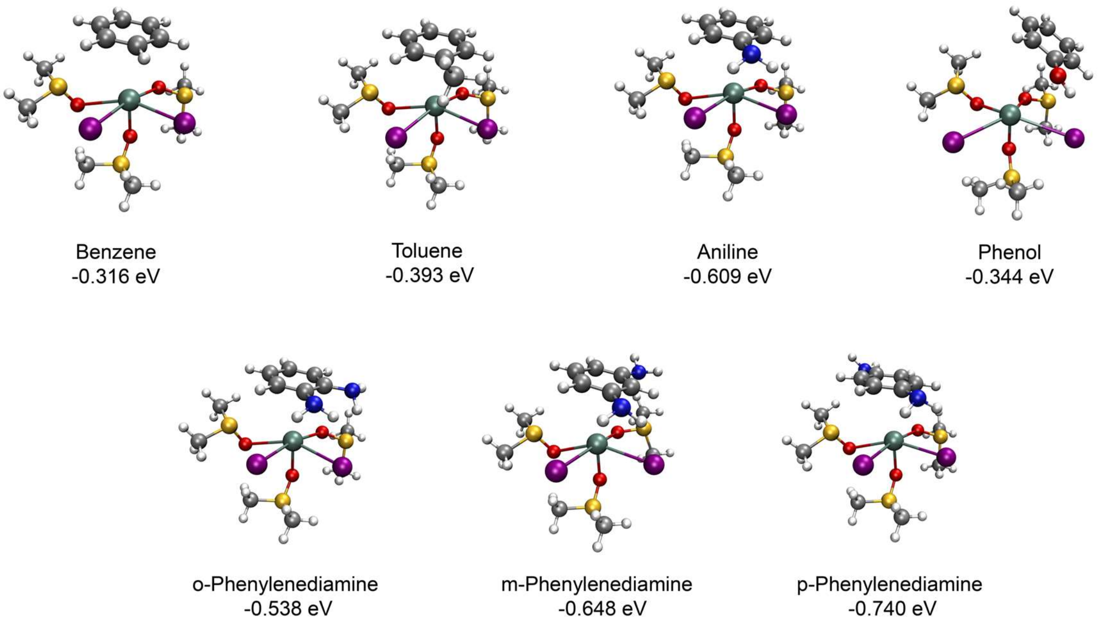

Supplementary Fig. 5. Complex structures, and binding energies of the molecules interacting with SnI_2•3DMSO.

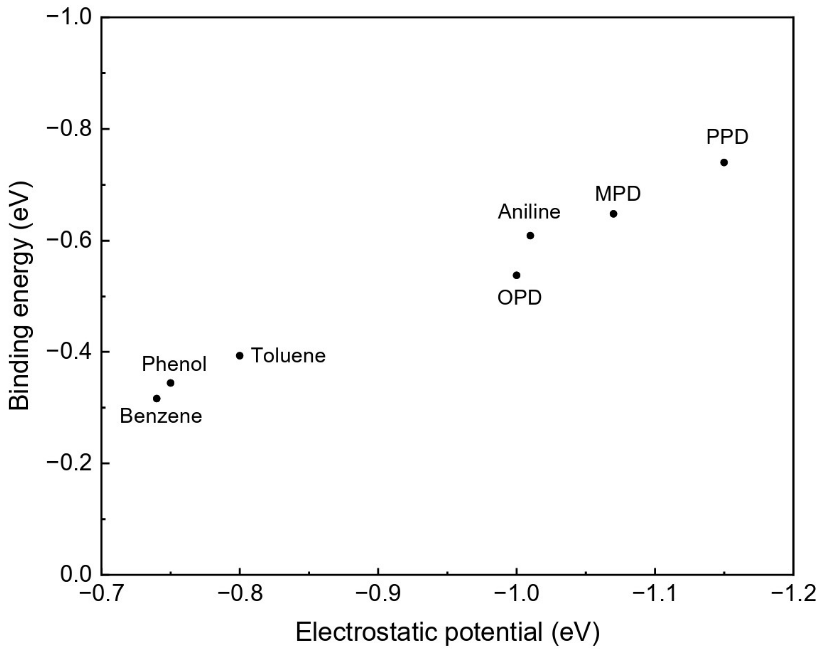

Supplementary Fig. 6. Relationship between the binding energy of the molecules with SnI_2•3DMSO and the local ESP minimum value near the ring center.

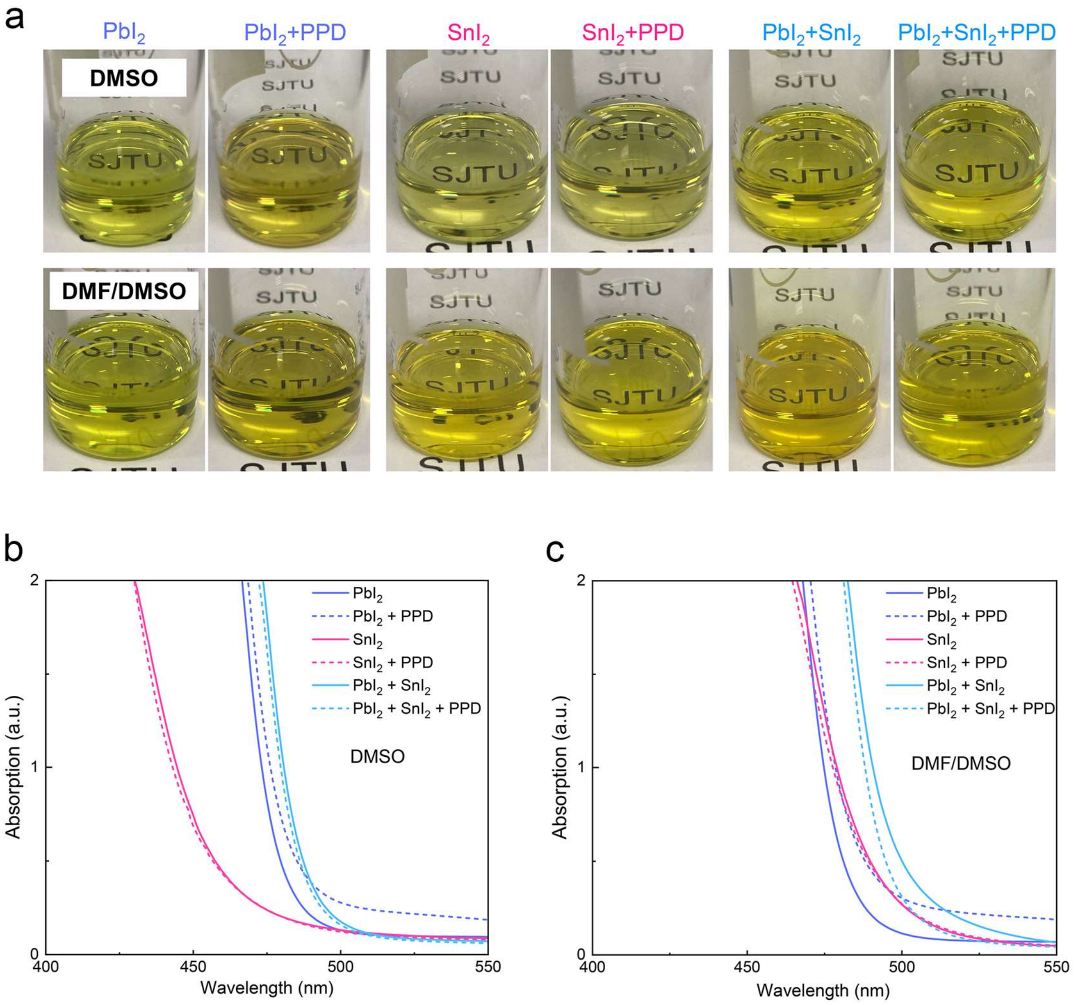

Supplementary Fig. 7. Interactions between PPD and PbI_2 and SnI_2. a, Photos of freshly dissolved PbI_2 (1 M), SnI_2 (1 M), and PbI_2+SnI_2 (0.5 M+0.5 M) without and with PPD additive (0.08 M). Upper row, DMSO solvent; row below, DMF/DMSO (v/v, 3:1) solvent. **b**, Absorption spectra of the DMSO-based fresh solutions. **c**, Absorption spectra of the DMF/DMSO-based fresh solutions.

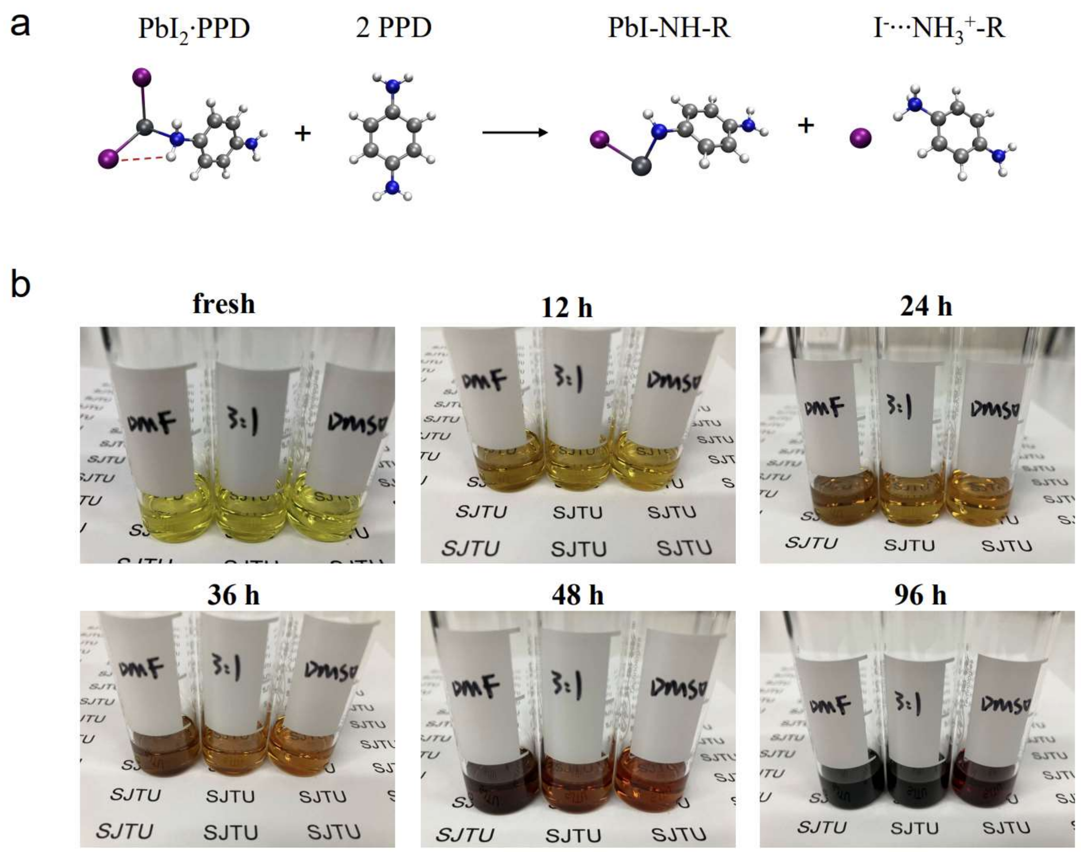

Supplementary Fig. 8. The acid-base reaction between PPD and PbI_2. a, The acid-base reaction mechanism between PPD and PbI_2. **b**, Photos of PbI_2+PPD (0.5 M+0.04 M) in DMF, DMF/DMSO (3:1, v/v), and DMSO, the solutions were sealed and stored in air for 96 hours.

Supplementary Note 1. Interactions between PPD and SnI_4 and raw SnI_2

The PPD agent was mixed into SnI_4 and raw SnI_2 solutions (Supplementary Fig. 8). The SnI_4 solution turned dark immediately upon incorporation of PPD, indicating the occurrence of the acid-base reaction, rather than a redox reaction. Noteworthy, the raw SnI_2 (containing SnI_4 impurity) solution with PPD demonstrated a blueshifted absorption spectrum. This revealed that the PPD preferentially interacts with Sn(II) in the Sn(II)/Sn(IV) mixed solution.

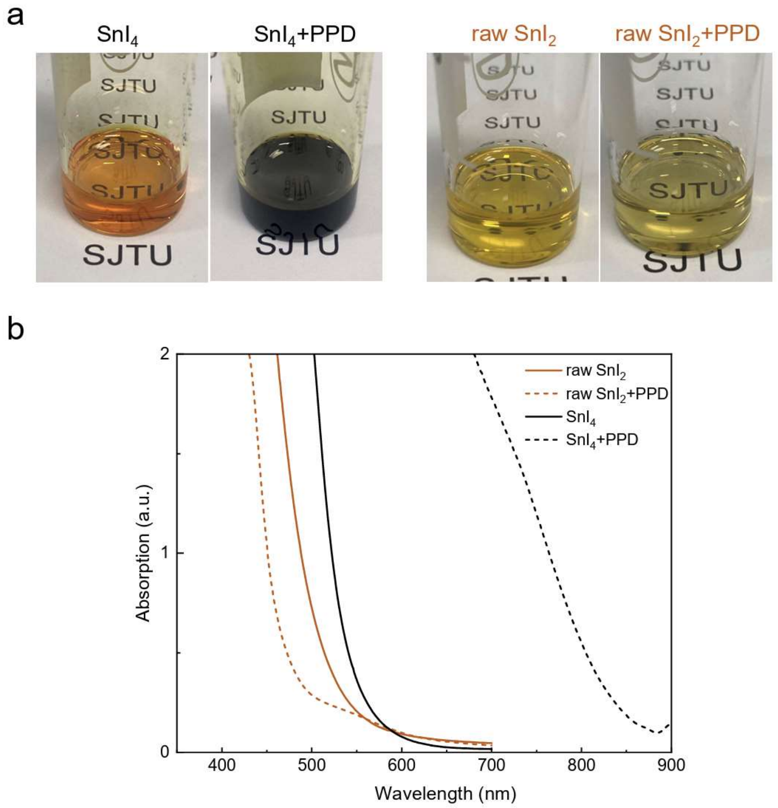

Supplementary Fig. 9. Interactions between PPD and SnI_4 and raw SnI_2. a, Photos of SnI_4 (0.1 M) and raw SnI_2 (1 M) dissolved in DMSO, with and without PPD (8 mol%). **b**, Absorption spectra of the above solutions.

Supplementary Note 2. Additional NMR analysis

The NMR spectra of PPD, PbI_2+PPD, SnI_2+PPD, and SnI_2+PbI_2+PPD in mixed DMF-d_7/DMSO-d_6 (3:1, v/v) are shown in Supplementary Fig. 10. In PPD+PbI_2, evident protonated PPD signals and a broadened -NH_2 peak are observed, revealing an enhanced extent of the Pb(II)-PPD dative bond and the acid-base reaction, due to the lower coordinating ability of DMF. In PPD+SnI_2 and PPD+PbI_2+SnI, no signal of protonated PPD was observed, and the deshielding of PPD protons became more pronounced than in the neat DMSO case, suggesting stronger interactions between PPD and the Sn(II) solvates.

We further conducted NMR measurements on FAI, FAI+PPD, $FAPbI_3$+PPD, $FASnI_3$+PPD, and $FAPb_{0.5}Sn_{0.5}I_3$+PPD (Supplementary Fig. 11). The broadened NH_2 signals in FAI+PPD reveal the intermolecular H-bonds. The spectrum of $FAPbI_3$+PPD is highly similar to that of FAI+PPD, implying that PPD molecules primarily interact with FA cations in the $FAPbI_3$ precursor. In contrast, for $FASnI_3$+PPD and $FAPb_{0.5}Sn_{0.5}I_3$+PPD, apart from the peak broadening, the PPD protons also present large downfield shifts, as they did in the SnI_2+PPD case. This observation demonstrates that the PPD-SnI_2 interactions remain in the perovskite precursor, coexisting with the FA-PPD H-bonds in the solution.

We further collected the ^{119}Sn NMR spectra of SnI_2, SnI_2+PPD, and PbI_2+SnI_2+PPD, and ^{207}Pb NMR spectra of PbI_2, PbI_2+PPD, and PbI_2+SnI_2+PPD, all dissolved in DMSO-d_6 (Supplementary Fig. 11). Compared to SnI_2, SnI_2+PPD and PbI_2+SnI_2+PPD exhibit upfield shifts of 24 and 27 ppm, respectively, as the electron-rich PPD molecule

enhances the screening of Sn(II). Compared to ^{119}Sn, ^{207}Pb has less NMR sensitivity and yields broad signals. The PbI_2, PbI_2+PPD, and PbI_2+SnI_2+PPD samples show chemical shift at -56, -61, and -59 ppm, respectively, the difference between which is negligible given the wide chemical shift range of ^{207}Pb. The results confirm that most Pb(II) cations do not interact with PPD in the solution.

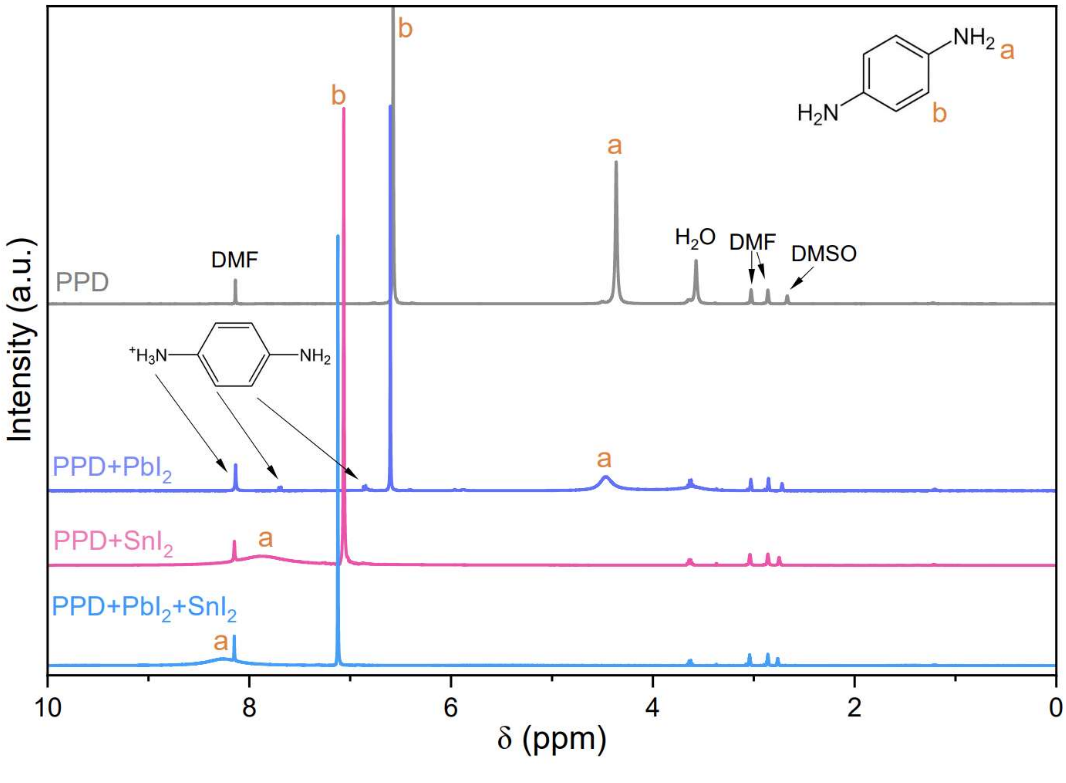

Supplementary Fig. 10. ^{1}H NMR spectra of PPD, PbI_2+PPD, SnI_2+PPD, and SnI_2+PbI_2+PPD in DMF-d_7/DMSO-d_6 (3:1, v/v).

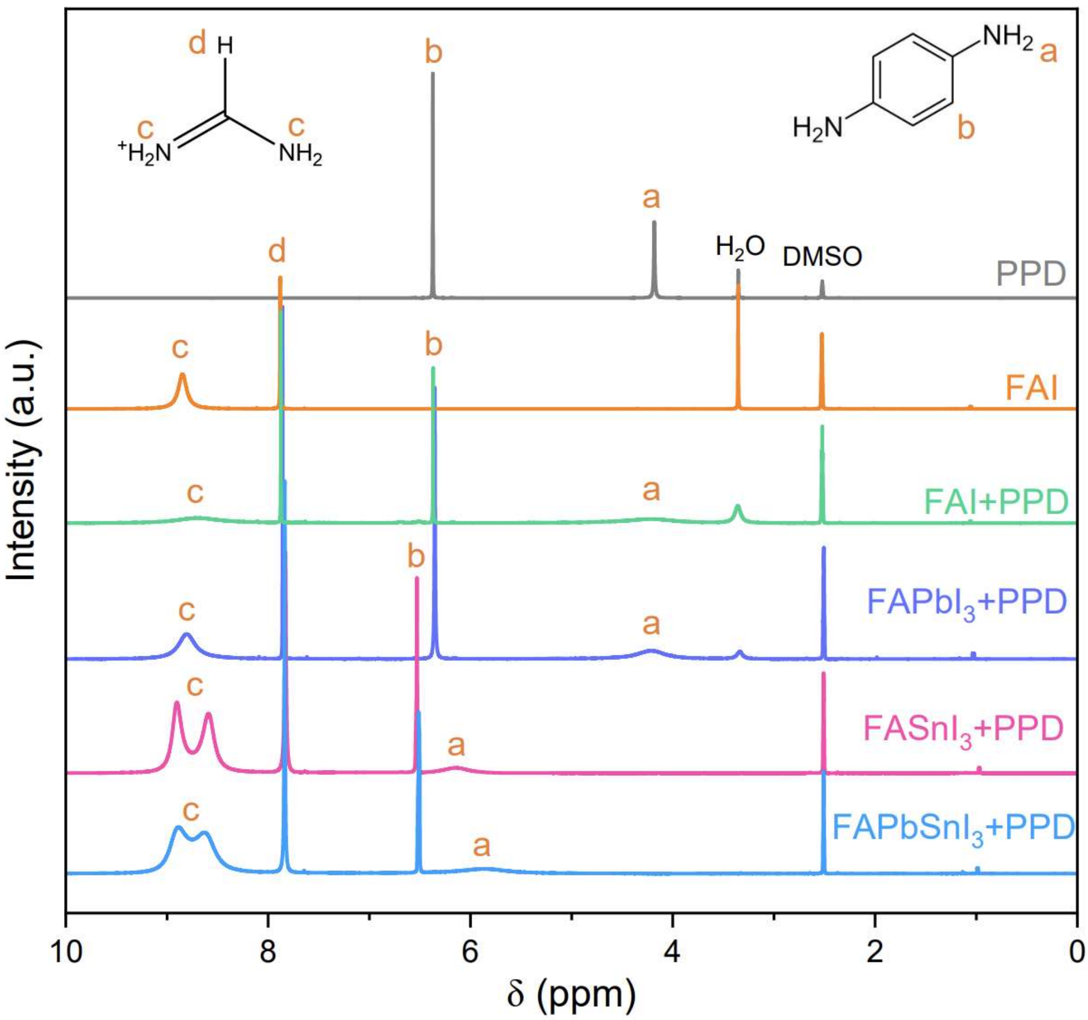

Supplementary Fig. 11. ^{1}H NMR spectra of PPD, FAI, FAI+PPD, $FAPbI_3$+PPD, $FASnI_3$+PPD, and $FAPb_{0.5}Sn_{0.5}I_3$+PPD in DMSO-d_6.

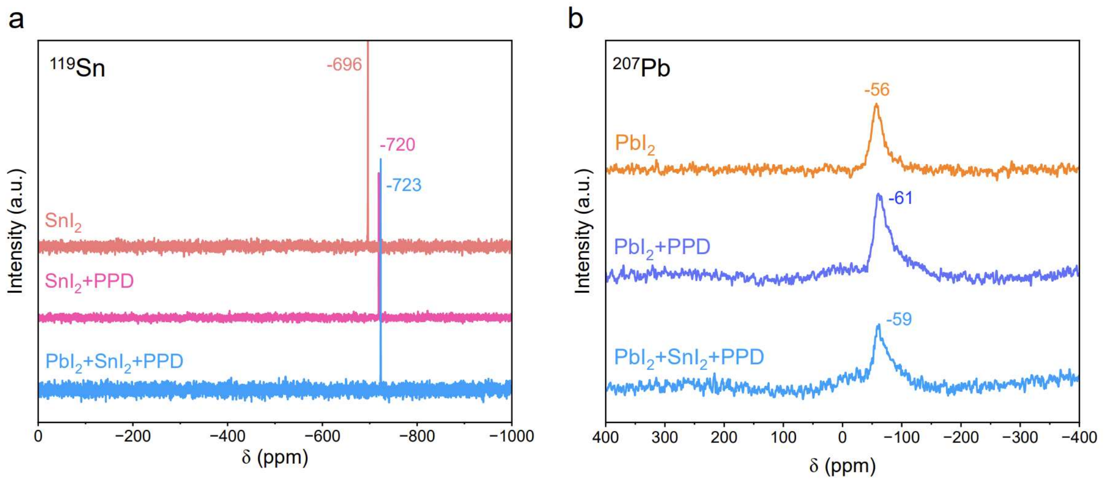

Supplementary Fig. 12. ^{119}Sn and ^{207}Pb NMR. a, ^{119}Sn NMR spectra of SnI_2, SnI_2+PPD, PbI_2+SnI_2+PPD in dmso-d_6; **b,** ^{207}Pb NMR spectra of PbI_2, PbI_2+PPD, PbI_2+SnI_2+PPD in dmso-d_6.

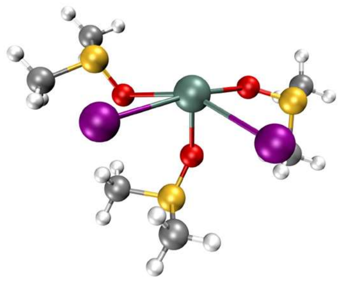

Bond	Distance (Å)
Sn-O	2.24
Sn-O	2.50
Sn-O	2.84
Sn-I	2.94
Sn-I	3.09

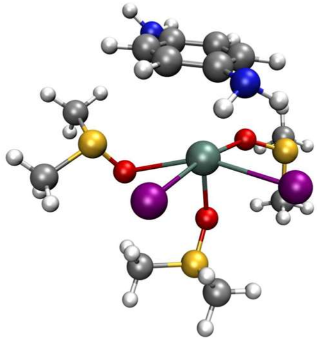

Bond	Distance (Å)
Sn-O	2.26
Sn-O	2.57
Sn-O	2.76
Sn-I	3.00
Sn-I	3.16
Sn-C	3.28
Sn-C	3.29
Sn-C	3.54
Sn-C	3.53
Sn-C	3.76
Sn-C	3.76
Sn-N	3.87
Sn-N	4.62

Supplementary Fig. 13. Bond lengths of SnI_2•3DMSO (left) and SnI_2•3DMSO•PPD (right) complexes.

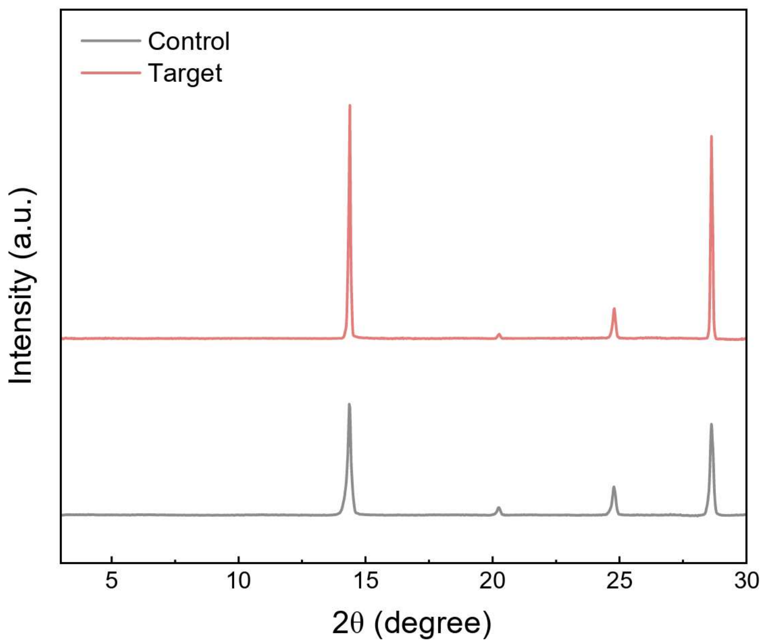

Supplementary Fig. 14. XRD patterns of the control and target perovskite films.

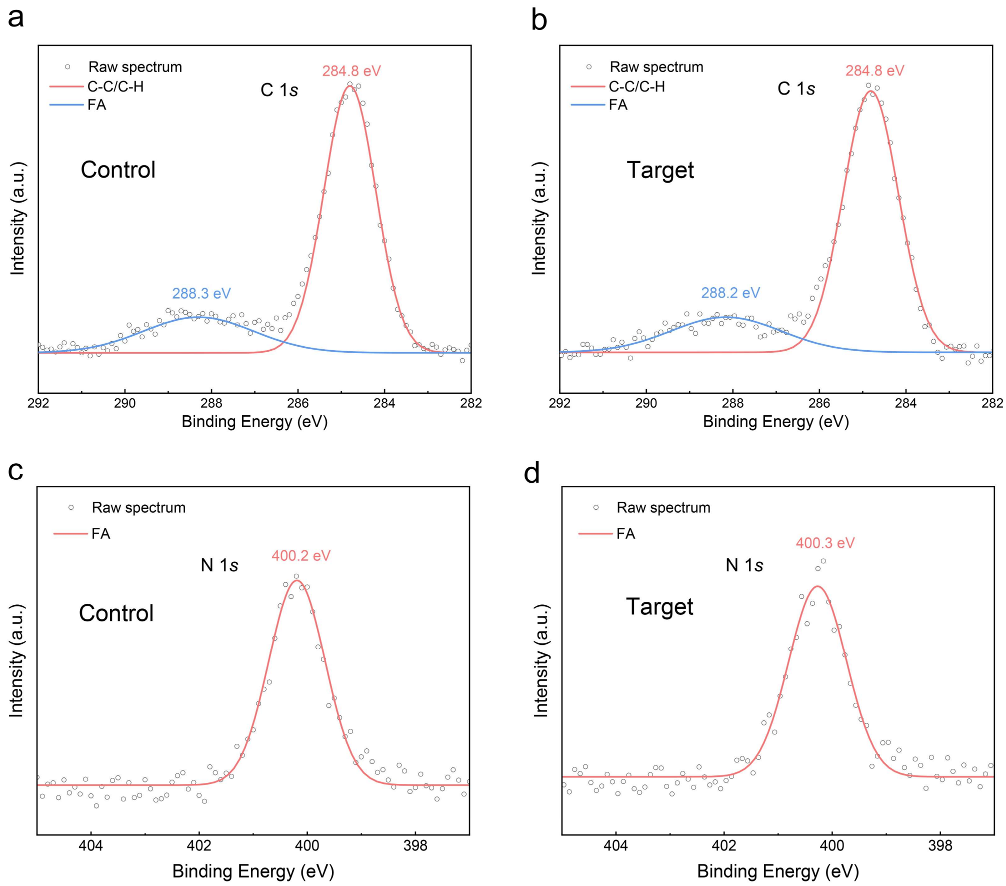

Supplementary Fig. 15. XPS spectra of control and target perovskite films. The XPS C 1*s* and N 1*s* core levels spectra of the control (**a, c**) and target perovskite films (**b, d**).

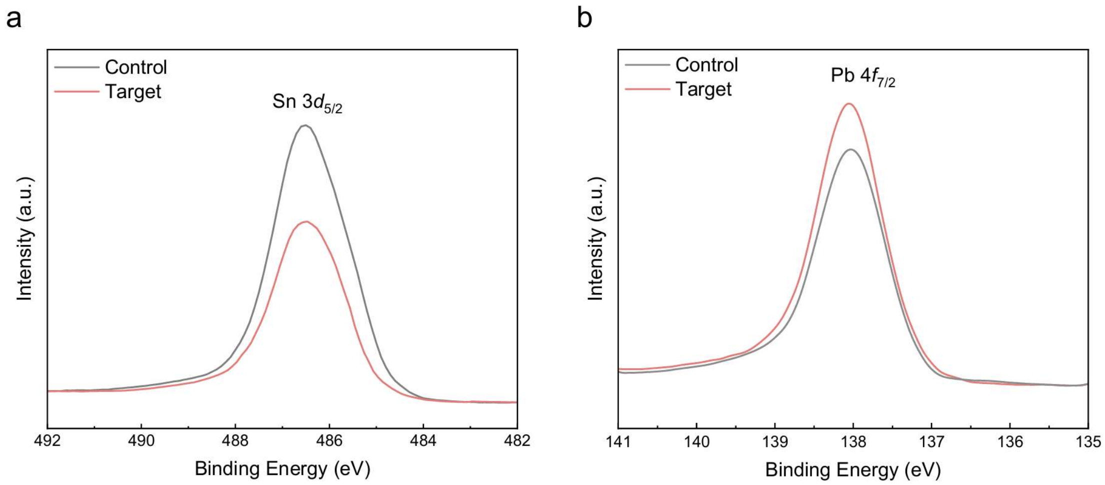

Supplementary Fig. 16. XPS spectra of the control and target perovskite films. a, Sn 3$d_{5/2}$ core-level; **b**, Pb 4$f_{7/2}$ core-level.

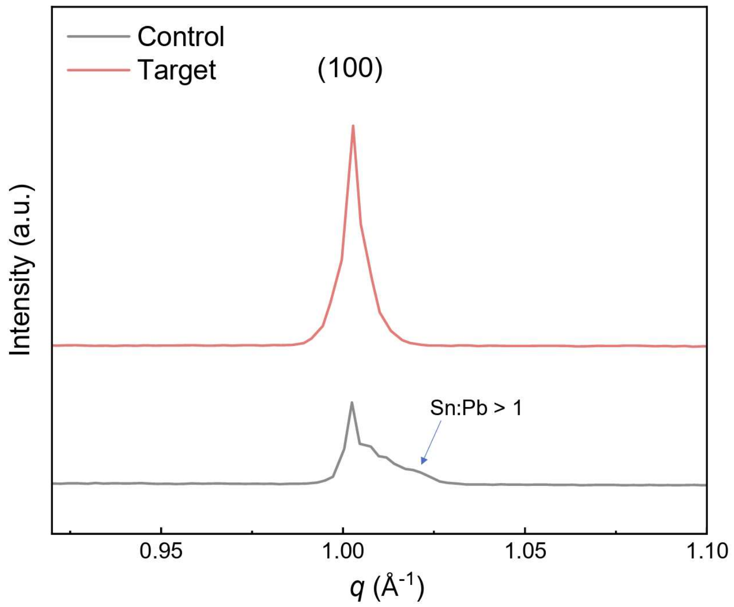

Supplementary Fig. 17. Out-of-plane linecut profiles of the GIWAXS patterns.

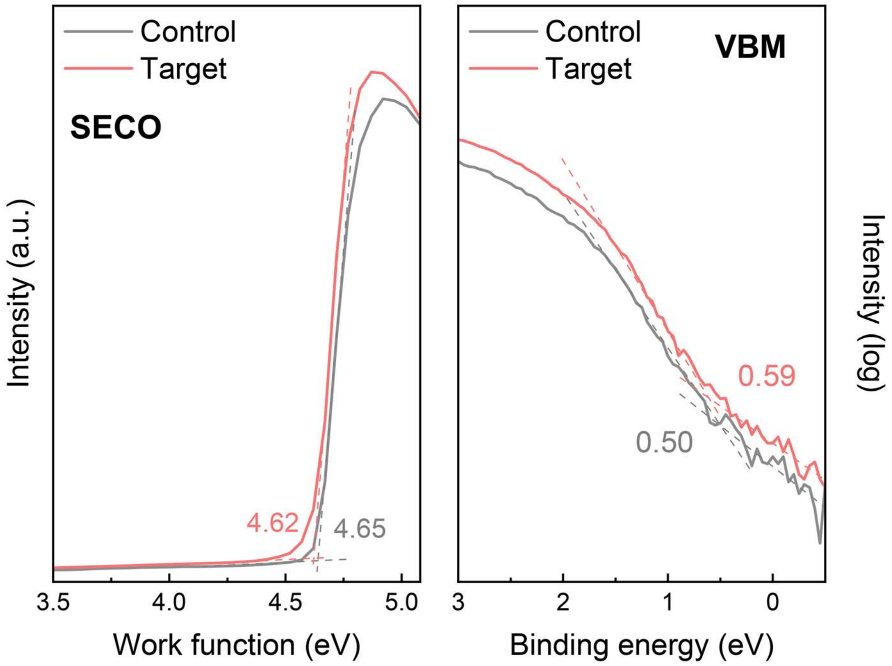

Supplementary Fig. 18. The UPS spectra of the control and target perovskite films. Left, secondary electron cut-off (SECO) region; right, valence band region.

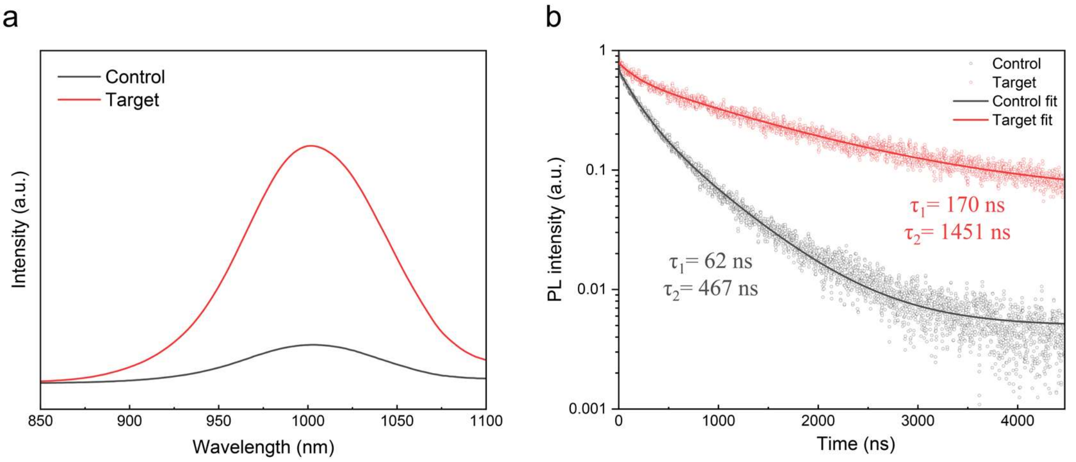

Supplementary Fig. 19. PL and TRPL spectra of control and target perovskite films. a, Steady-state PL spectra of the control and target perovskite films. **b**, Time-resolved PL spectra of the control and target perovskite films. The lifetimes were obtained through fitting by the equation $y = y_0 + A_1 e^{\frac{-t}{\tau_1}} + A_2 e^{\frac{-t}{\tau_2}}$.

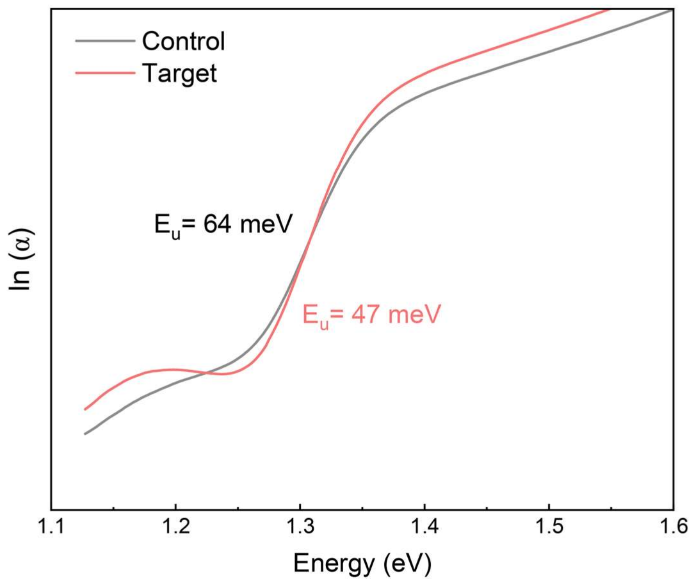

Supplementary Fig. 20. The Urbach energies of the control and target perovskite films, derived from absorption spectra.

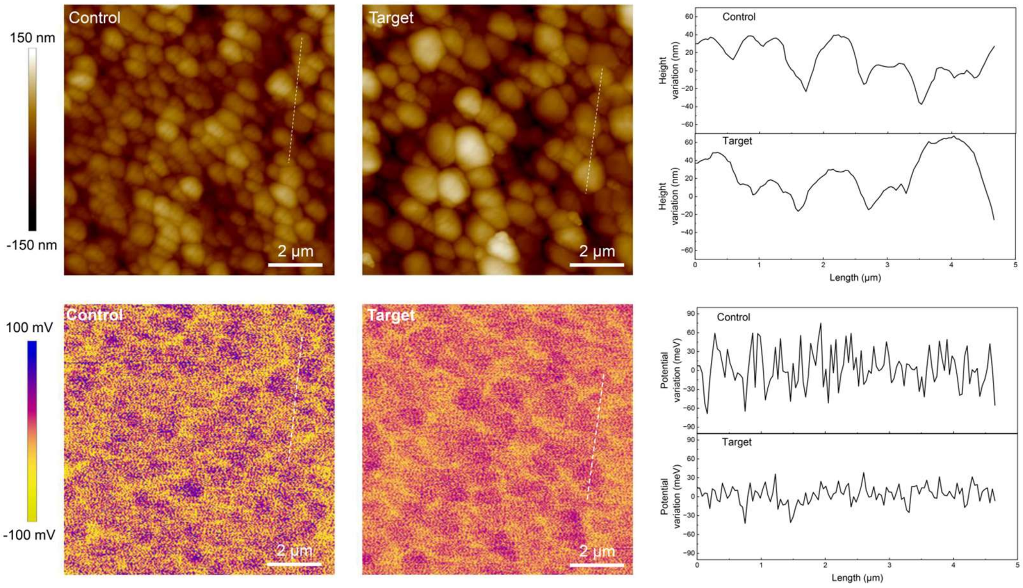

Supplementary Fig. 21. AFM surface morphology (up) and surface potential distribution (below) of the perovskite films.

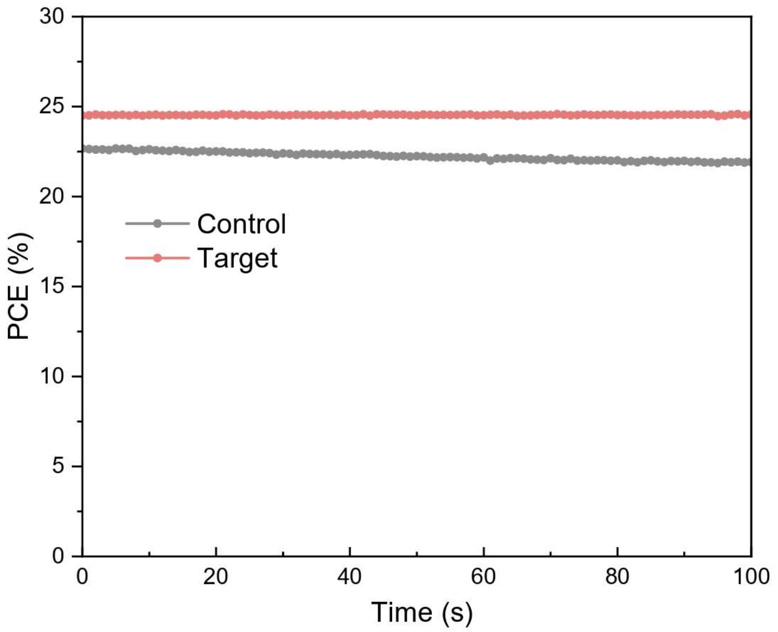

Supplementary Fig. 22. The steady-state power output at the maximum power point over 100 seconds.

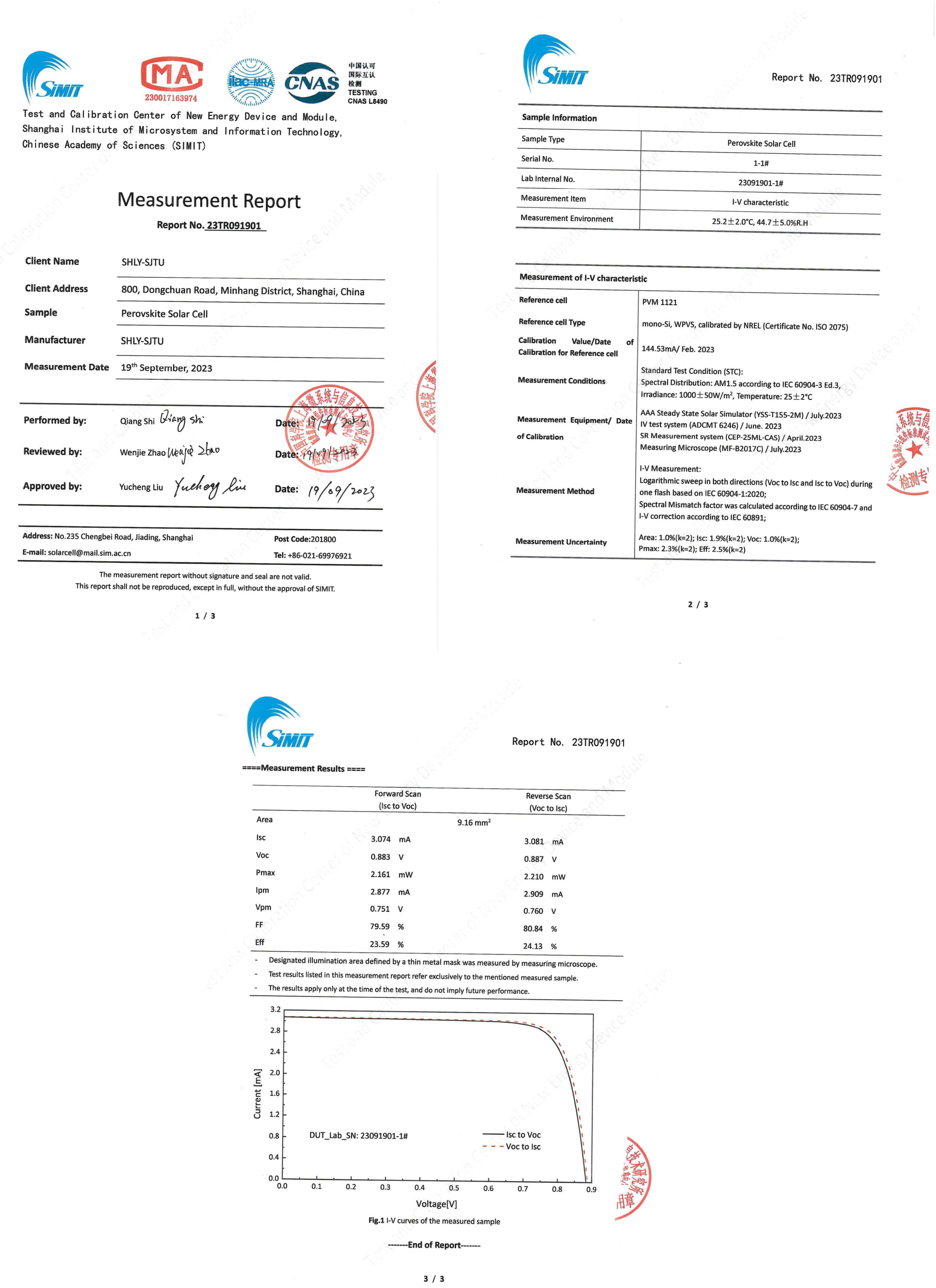

中国认可
国际互认
检测
TESTING
CNAS L8490

230017163974

Test and Calibration Center of New Energy Device and Module,
Shanghai Institute of Microsystem and Information Technology,
Chinese Academy of Sciences (SIMIT)

Measurement Report

Report No. 23TR091901

**Client Name**	SHLY-SJTU
**Client Address**	800, Dongchuan Road, Minhang District, Shanghai, China
**Sample**	Perovskite Solar Cell
**Manufacturer**	SHLY-SJTU
**Measurement Date**	19th September, 2023

**Performed by:**	Qiang Shi Qiang Shi	**Date:** 19/09/2023
**Reviewed by:**	Wenjie Zhao Wenjie Zhao	**Date:** 19/09/2023
**Approved by:**	Yucheng Liu Yucheng Liu	**Date:** 19/09/2023

Address: No.235 Chengbei Road, Jiading, Shanghai **Post Code:**201800
E-mail: solarcell@mail.sim.ac.cn **Tel:** +86-021-69976921

The measurement report without signature and seal are not valid.
This report shall not be reproduced, except in full, without the approval of SIMIT.

1 / 3

Report No. 23TR091901

Sample Information

Sample Type	Perovskite Solar Cell
Serial No.	1-1#
Lab Internal No.	23091901-1#
Measurement Item	I-V characteristic
Measurement Environment	25.2±2.0°C, 44.7±5.0%R.H

Measurement of I-V characteristic

**Reference cell**	PVM 1121
**Reference cell Type**	mono-Si, WPVS, calibrated by NREL (Certificate No. ISO 2075)
**Calibration Value/Date of Calibration for Reference cell**	144.53mA/ Feb. 2023
**Measurement Conditions**	Standard Test Condition (STC):<br>Spectral Distribution: AM1.5 according to IEC 60904-3 Ed.3,<br>Irradiance: 1000±50W/m², Temperature: 25±2°C
**Measurement Equipment/ Date of Calibration**	AAA Steady State Solar Simulator (YSS-T155-2M) / July.2023<br>IV test system (ADCMT 6246) / June. 2023<br>SR Measurement system (CEP-25ML-CAS) / April.2023<br>Measuring Microscope (MF-B2017C) / July.2023
**Measurement Method**	I-V Measurement:<br>Logarithmic sweep in both directions (Voc to Isc and Isc to Voc) during one flash based on IEC 60904-1:2020;<br>Spectral Mismatch factor was calculated according to IEC 60904-7 and I-V correction according to IEC 60891;
**Measurement Uncertainty**	Area: 1.0%(k=2); Isc: 1.9%(k=2); Voc: 1.0%(k=2);<br>Pmax: 2.3%(k=2); Eff: 2.5%(k=2)

2 / 3

Report No. 23TR091901

====Measurement Results ====

	Forward Scan (Isc to Voc)		Reverse Scan (Voc to Isc)	
Area		9.16 mm²		
Isc	3.074	mA	3.081	mA
Voc	0.883	V	0.887	V
Pmax	2.161	mW	2.210	mW
Ipm	2.877	mA	2.909	mA
Vpm	0.751	V	0.760	V
FF	79.59	%	80.84	%
Eff	23.59	%	24.13	%

- Designated illumination area defined by a thin metal mask was measured by measuring microscope.
- Test results listed in this measurement report refer exclusively to the mentioned measured sample.
- The results apply only at the time of the test, and do not imply future performance.

Fig.1 I-V curves of the measured sample

-------End of Report-------

3 / 3

Supplementary Fig. 23. The certified result of a small-area (0.0916 cm^2) Sn-Pb perovskite solar cell, measured at the Test and Calibration Centre of the New Energy Device and Module, SIMIT, Chinese Academy of Sciences.

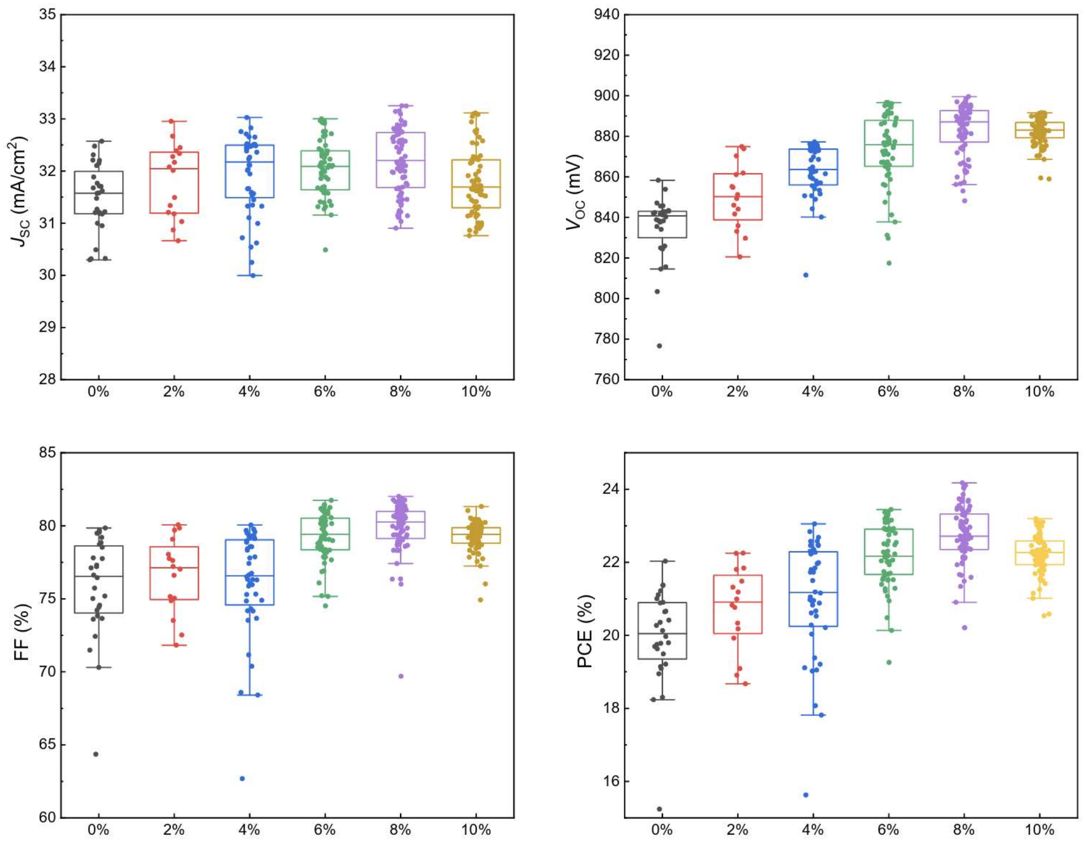

Supplementary Fig. 24. Statistics of device parameters of different PPD concentrations. Center line, median; box range, 25/75 percentiles; whiskers, the outermost outliers within 1.5 × interquartile range. The number of the 0%, 2%, 4%, 6%, 8%, and 10% cells tested was 28, 16, 44, 60, 80, and 72, respectively, collected from 6 different batches.

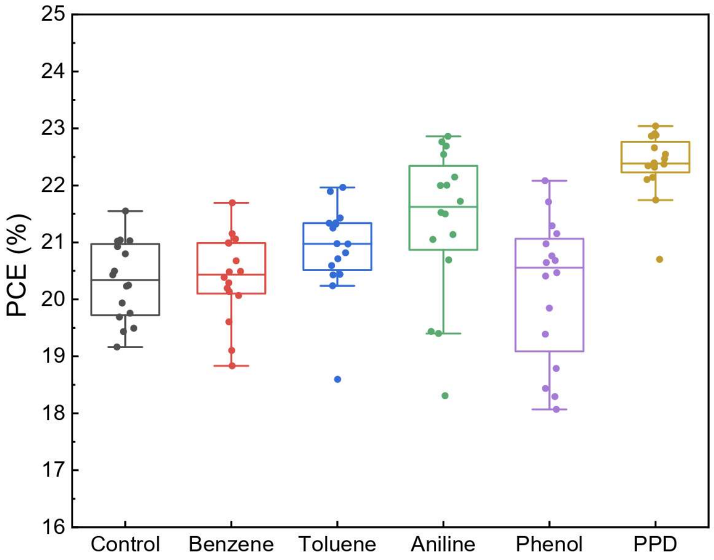

Supplementary Fig. 25. The PCE statistic of devices incorporating the different additives; the doping concentration is 4% (molar ratio with respect to SnI_2). Center line, median; box range, 25/75 percentiles; whiskers, the outermost outliers within 1.5 × interquartile range.

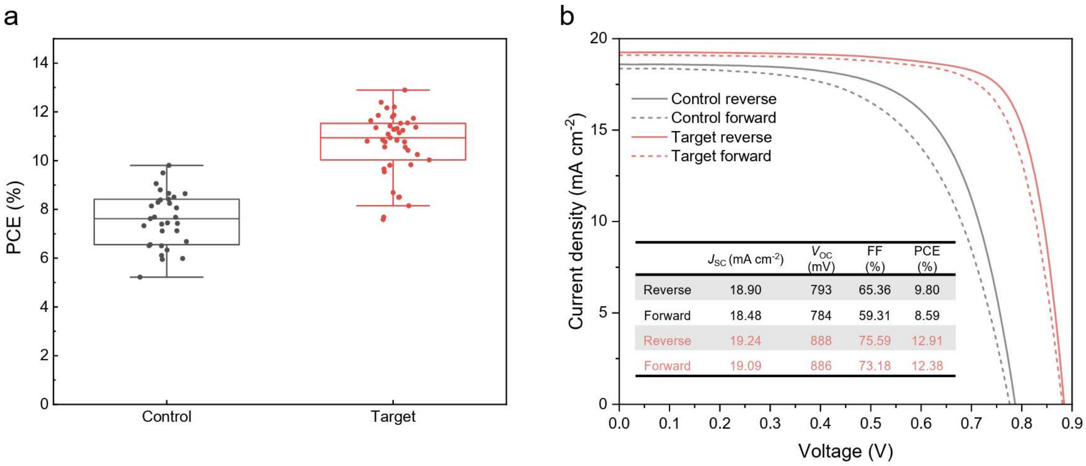

Supplementary Fig. 26. Device performance of Sn-based PSCs. PCE statistic (**a**) and *J*-*V* curves (**b**) of Sn-based perovskite solar cells. Center line, median; box range, 25/75 percentiles; whiskers, the outermost outliers within 1.5 × interquartile range.

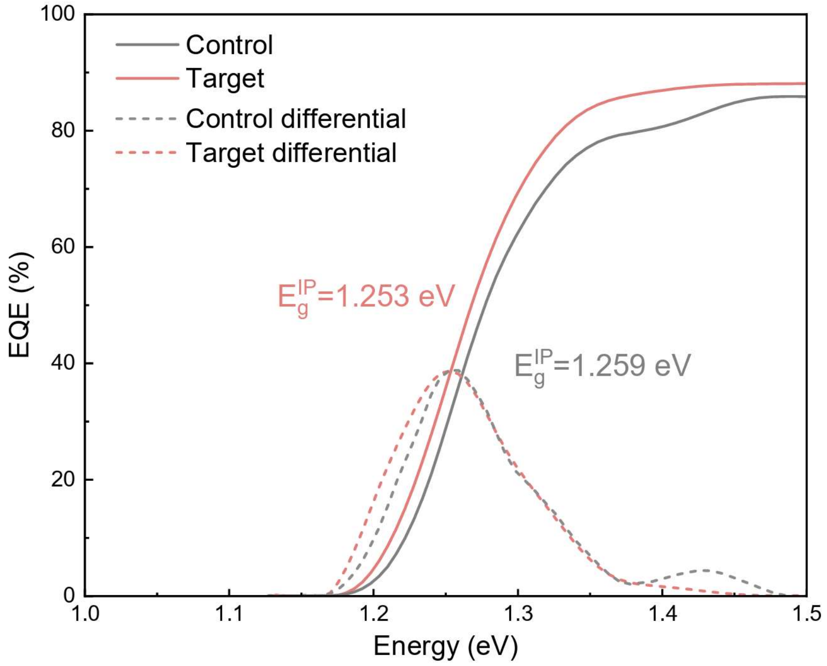

Supplementary Fig. 27. Bandgap determination of the perovskite absorber based on the EQE data.

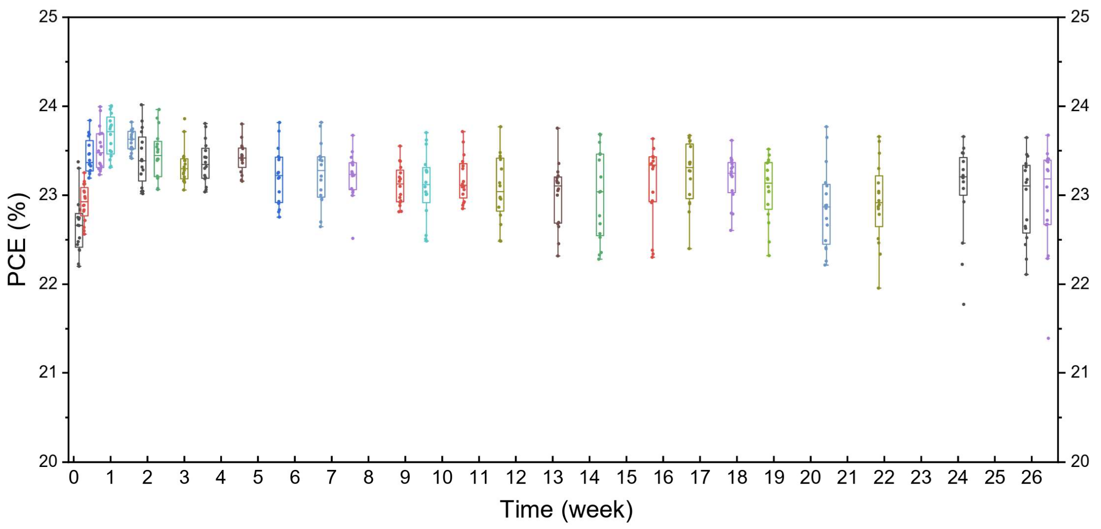

Supplementary Fig. 28. The shelf stability of 16 unencapsulated cells during six months (dark; N_2). Center line, median; box range, 25/75 percentiles; whiskers, the outermost outliers within 1.5 × interquartile range.

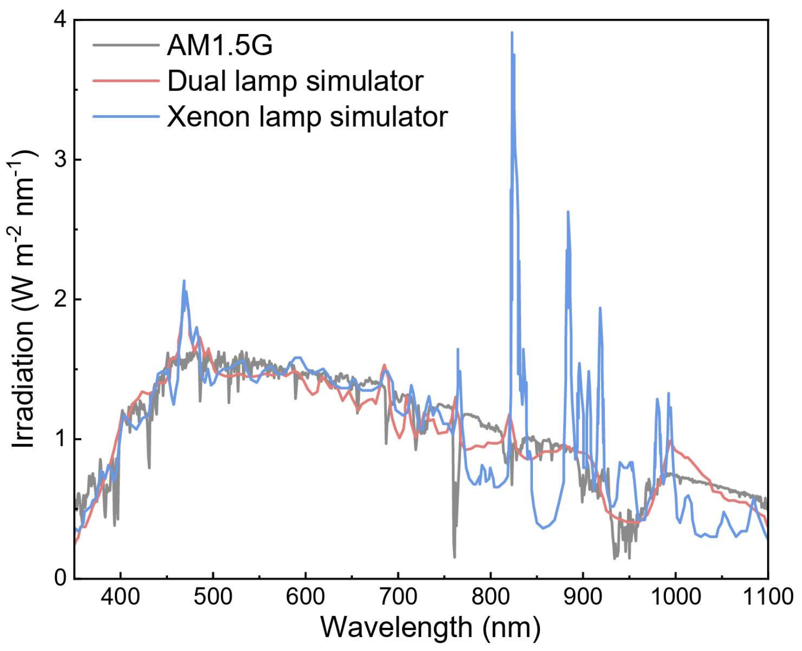

Supplementary Fig. 29. Spectra of the dual lamp simulator (for efficiency measurement) and the Xenon lamp simulator (for MPP tracking).

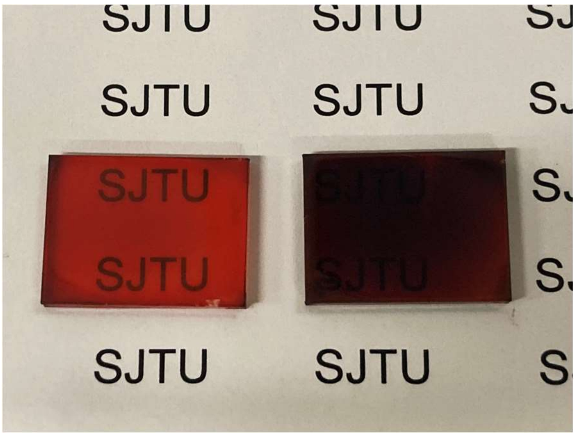

Supplementary Fig. 30. Photos of bare control (left) and target (right) perovskite films kept in the air (RH 30–40%, temperature 20–30 °C) for about ~90 hours.

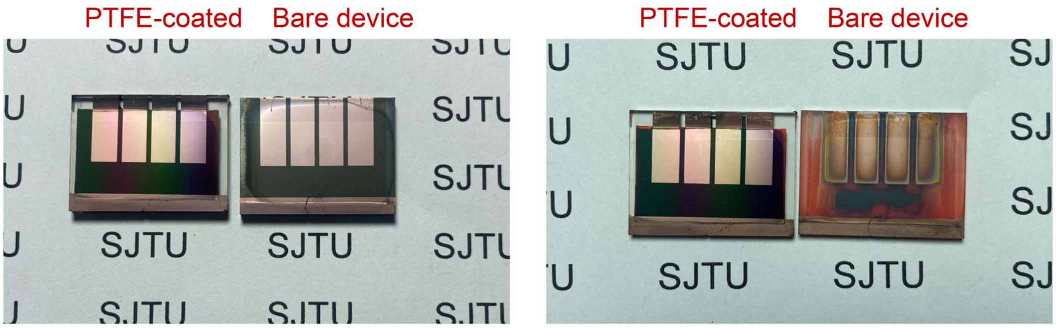

Supplementary Fig. 31. Photos of devices stored in the air (RH 30–40%, temperature 20–30 ℃), with or without a PTFE coating layer. Left, fresh devices; right, devices aged for 15 days.

Supplementary Table 1. Components of interaction energy between PPD and SnI_2·3DMSO through SAPT calculation.

Component	Energy (eV)
Electrostatic	-0.8879
Exchange repulsion	1.2867
Induction	-0.1790
Charge-transfer	-0.0390
Dispersion	-0.9126
Total	-0.7318

Supplementary Table 2. Summaries of stability performances of single-junction Sn-Pb perovskite solar cells.

Device structure	PCE	Test conditions	Stability	Ref
ITO/PEDOT:PSS/perovskite/PCBM/BCP/Ag	22.4%	MPPT in N_2, 25 °C	98.7% for 150 h	[1]
ITO/P3CT-Cs/perovskite/C_{60}/BCP/Ag	23.2%	N_2, 65 °C storage	90% for 300 h	[2]
ITO/PEDOT:PSS/perovskite/C_{60}/SnO_2/Ag	23.4%	MPPT in N_2, 35 °C	85% for 1000 h	[3]
ITO/PEDOT/perovskite/BDA/PCBM/C_{60} /SnO_2/Cu	23.7%	MPPT in air, 50±5 °C, Encapsulation	90% for 906 h 88% for 1000 h	[4]
ITO/PEDOT:PSS/perovskite/C_{60}/BCP/Cu	22.2%	N_2, RT storage	100% for 2300 h	[5]
ITO/PEDOT:PSS/perovskite/C_{60}/BCP/Ag	22.7%	MPPT in N_2, 45 °C	80% for 400 h	[6]
ITO/SnOCl/perovskite/BDA/PCBM/C_{60} /SnO_2/Cu	22.2%	One sun in air, ~50 °C, Encapsulation	81% for 850 h	[7]
FTO/PEDOT:PSS/perovskite/C_{60}/BCP/Cu	22.7%	N_2, dark, RT storage	96% for 3 months	[8]
ITO/PEDOT:PSS/perovskite/C_{60}/BCP/Cu	23.1%	N_2, RT storage	97.5% for 3500 h	[9]
FTO/PEDOT:PSS/perovskite/C_{60}/BCP/Cu	23.6%	MPPT in N_2	80% for 200 h	[10]
ITO/PEDOT:PSS/perovskite/C_{60}/BCP/Ag	22.1%	MPPT in N_2, 30-35°C	82% for 1830 h	[11]
FTO/2PACz-MPA/perovskite/ PCBM/C_{60}/BCP/Ag	23.3%	One sun in N_2, RT	90% for 1026 h	[12]
ITO/PEDOT:PSS/perovskite/C_{60}/SnO_2/Ag	23.4%	MPPT in N_2	90% for 1000 h	[13]
FTO/PEDOT:PSS/perovskite/C_{60}/BCP/Cu	24.5%	MPPT in air, 20-30 °C, Encapsulation	90% for 795 h	This work

Supplementary References

1. Zhang, Z. *et al.* DMSO-Free Solvent Strategy for Stable and Efficient Methylammonium‐Free Sn-Pb Alloyed Perovskite Solar Cells. *Advanced Energy Materials*, 2300181 (2023).
2. Zhang, W. *et al.* Component Distribution Regulation in Sn-Pb Perovskite Solar Cells through Selective Molecular Interaction. *Adv. Mater.* **35** (2023).
3. Yan, W. *et al.* Hot‐Carrier Cooling Regulation for Mixed Sn-Pb Perovskite Solar Cells. *Adv. Mater.* **36** (2024).
4. Wang, J. *et al.* Enhancing Photostability of Sn-Pb Perovskite Solar Cells by an Alkylammonium Pseudo-Halogen Additive. *Advanced Energy Materials* **13**, 2204115 (2023).
5. Huang, L. *et al.* Efficient Narrow-bandgap Mixed Tin-lead Perovskite Solar Cells via Natural Tin Oxide Doping. *Adv. Mater.* (2023).
6. Chen, L. *et al.* Incorporating Potassium Citrate to Improve the Performance of Tin-Lead Perovskite Solar Cells. *Advanced Energy Materials* (2023).
7. Yu, Z. *et al.* Solution‐Processed Ternary Tin (II) Alloy as Hole-Transport Layer of Sn‐Pb Perovskite Solar Cells for Enhanced Efficiency and Stability. *Adv. Mater.* **34**, 2205769 (2022).
8. Hu, S. *et al.* Synergistic Surface Modification of Tin-Lead Perovskite Solar Cells. *Adv. Mater.*, 2208320 (2022).
9. Zhou, J. *et al.* Mixed tin-lead perovskites with balanced crystallization and oxidation barrier for all-perovskite tandem solar cells. *Nature Communications* **15** (2024).
10. Hu, S. *et al.* Optimized carrier extraction at interfaces for 23.6% efficient tin-lead perovskite solar cells. *Energy Environ. Sci.* **15**, 2096-2107 (2022).
11. Tong, J. *et al.* Carrier control in Sn-Pb perovskites via 2D cation engineering for all-perovskite tandem solar cells with improved efficiency and stability. *Nat. Energy* **7**, 642–651 (2022).
12. Kapil, G. *et al.* Tin–Lead Perovskite Solar Cells Fabricated on Hole Selective Monolayers. *ACS Energy Letters* **7**, 966-974 (2022).
13. Tan, S. *et al.* Sustainable thermal regulation improves stability and efficiency in all-perovskite tandem solar cells. *Nature Communications* **15** (2024).
