# Supplementary material for: Synchronized crystallization in tin-lead perovskite solar cells
Source: Nat Commun. 2024 Aug 12;15:6887. doi: 10.1038/s41467-024-51361-2 (PMC11319464; doi:10.1038/s41467-024-51361-2)
Supplement: Supplementary file 3 — Description of Additional Supplementary Files [file 41467_2024_51361_MOESM3_ESM.pdf]

Supplementary Movie 1.
AIMD simulation of PPD interacting with $SnI_2 \cdot 3DMSO$ under 300 K for 3 μs.

Supplementary Movie 2.
AIMD simulation of PPD interacting with $PbI_2 \cdot DMSO$ under 300 K for 3 μs.

Supplementary Movie 3.
AIMD simulation of PPD interacting with $PbI_2 \cdot 2DMSO$ under 300 K for 3 μs.
