# Supplementary material for: Synchronized crystallization in tin-lead perovskite solar cells
Source: Nat Commun. 2024 Aug 12;15:6887. doi: 10.1038/s41467-024-51361-2 (PMC11319464; doi:10.1038/s41467-024-51361-2)
Supplement: Supplementary file 7 — Reporting Summary [file 41467_2024_51361_MOESM7_ESM.pdf]

nature portfolio

Corresponding author(s): Han Chen

Solar Cells Reporting Summary

Nature Portfolio wishes to improve the reproducibility of the work that we publish. This form is intended for publication with all accepted papers reporting the characterization of photovoltaic devices and provides structure for consistency and transparency in reporting. Some list items might not apply to an individual manuscript, but all fields must be completed for clarity.

For further information on Nature Research policies, including our data availability policy, see Authors & Referees.

Experimental design

Please check the following details are reported in the manuscript, and provide a brief description or explanation where applicable.

1. Dimensions

Item	Yes/No	Description / Explanation
Area of the tested solar cells	[x] Yes [ ] No	The active area is 0.0916 square centimeters.
Method used to determine the device area	[x] Yes [ ] No	The device area was defined by a metal aperture. See 'Method' section, 'Solar cell Characterizations' subsection.

2. Current-voltage characterization

Item	Yes/No	Description / Explanation
Current density-voltage (J-V) plots in both forward and backward direction	[x] Yes [ ] No	Fig. 4b in the manuscript.
Voltage scan conditions	[x] Yes [ ] No	J–V curves were measured in reverse scan (1.0 V to -0.1 V) and forward scan (-0.1 V to 1.0 V) under a constant scan speed of 100 mV/s with voltage steps of 10 mV and a delay time of 100 ms.
Test environment	[x] Yes [ ] No	The measurements were conducted in dry air (RH 10–20%, environment temperature 20–30 ℃).
Protocol for preconditioning of the device before its characterization	[x] Yes [ ] No	No preconditioning was performed.
Stability of the J-V characteristic	[x] Yes [ ] No	Efficiency near the maximum power point was record for 100 seconds. See Supplementary Fig. 22

3. Hysteresis or any other unusual behaviour

Item	Yes/No	Description / Explanation
Description of the unusual behaviour observed during the characterization	[x] Yes [ ] No	A small hysteresis (1.3%) was observed.
Related experimental data	[x] Yes [ ] No	J-V curves and parameters under reverse and forward scans were provided in Fig. 4b.

4. Efficiency

Item	Yes/No	Description / Explanation
External quantum efficiency (EQE) or incident photons to current efficiency (IPCE)	[x] Yes [ ] No	EQE curves were provided in Fig. 4c.
A comparison between the integrated response under the standard reference spectrum and the response measure under the simulator	[x] Yes [ ] No	The integrated photocurrent density can be found in Fig. 4c. The Jsc measured under the simulator can be found in Fig. 4b.

For tandem solar cells, the bias illumination and bias voltage used for each subcell

☐ Yes — *Provide a description of the measurement conditions.*
☒ No — No tandem cell is involved in this work.

5. Calibration

Light source and reference cell or sensor used for the characterization

☒ Yes — A dual light source simulator (WXS-90S-L2, Wacom) was used as the light source. A mono-Si reference cell (91150-KG5) is used for simulator calibration.
☐ No — *Explain why this information is not reported/not relevant.*

Confirmation that the reference cell was calibrated and certified

☒ Yes — The mono-Si reference cell was calibrated by Newport.
☐ No — *Explain why this information is not reported/not relevant.*

Calculation of spectral mismatch between the reference cell and the devices under test

☒ Yes — The spectral mismatch factor was controlled within 0.99–1 at the wavelength range of 350–1100 nm by tuning the simulator.
☐ No — *Explain why this information is not reported/not relevant.*

6. Mask/aperture

Size of the mask/aperture used during testing

☒ Yes — 0.0916 square centimeters.
☐ No — *Explain why this information is not reported/not relevant.*

Variation of the measured short-circuit current density with the mask/aperture area

☐ Yes — *Report the difference in the short-circuit current density values measured with the mask and aperture area.*
☒ No — All measurements were conducted using the same mask.

7. Performance certification

Identity of the independent certification laboratory that confirmed the photovoltaic performance

☒ Yes — Test and Calibration Centre of the New Energy Device and Module, SIMIT, Chinese Academy of Sciences, in Shanghai, China
☐ No — *Explain why this information is not reported/not relevant.*

A copy of any certificate(s)

☒ Yes — Supplementary Fig. 23.
☐ No — *Explain why this information is not reported/not relevant.*

8. Statistics

Number of solar cells tested

☒ Yes — We tested 80 cells (20 individual substrates) for the champion device.
☐ No — *Explain why this information is not reported/not relevant.*

Statistical analysis of the device performance

☒ Yes — Supplementary Fig. 24
☐ No — *Explain why this information is not reported/not relevant.*

9. Long-term stability analysis

Type of analysis, bias conditions and environmental conditions

☒ Yes — Maximum power point tracking of the encapsulated device was operated at the VMPP voltage (0.76 V) in ambient air (RH 30–40%, temperature 20–30 ℃) under simulated one-sun illumination (Xenon lamp; SS-X100R, Enlitech). See more details in the 'Method' section, the 'Devices encapsulation and stability test' subsection.
☐ No — *Explain why this information is not reported/not relevant.*
