# Supplementary material for: Synchronized crystallization in tin-lead perovskite solar cells
Source: Nat Commun. 2024 Aug 12;15:6887. doi: 10.1038/s41467-024-51361-2 (PMC11319464; doi:10.1038/s41467-024-51361-2)
Supplement: Supplementary file 2 — Peer Review File [file 41467_2024_51361_MOESM2_ESM.pdf]

nature portfolio

Peer Review File

Synchronized crystallization in tin-lead perovskite solar cells

Open Access This file is licensed under a Creative Commons Attribution 4.0 International License, which permits use, sharing, adaptation, distribution and reproduction in any medium or format, as long as you give appropriate credit to the original author(s) and the source, provide a link to the Creative Commons license, and indicate if changes were made. In the cases where the authors are anonymous, such as is the case for the reports of anonymous peer reviewers, author attribution should be to 'Anonymous Referee' followed by a clear attribution to the source work. The images or other third party material in this file are included in the article's Creative Commons license, unless indicated otherwise in a credit line to the material. If material is not included in the article's Creative Commons license and your intended use is not permitted by statutory regulation or exceeds the permitted use, you will need to obtain permission directly from the copyright holder. To view a copy of this license, visit http://creativecommons.org/licenses/by/4.0/.

Editorial Note: This manuscript has been previously reviewed at another journal that is not operating a transparent peer review scheme. This document only contains reviewer comments and rebuttal letters for versions considered at *Nature Communications*.

Reviewers' Comments:

Reviewer #1:
Remarks to the Author:
The authors propose the use of a non-covalent binding agent to control Sn-Pb perovskite crystallisation, and obtain excellent photovoltaic results. They clarified the point on the Sn lone pair as well as the confusing statements in the introduction part, and added interesting NMR findings. The reported data and discussion is consistent and it is of high scientific value for the perovskite community. I recommend this work in its current state for publication in Nature Communications.

Reviewer #3:
Remarks to the Author:
The authors have responded to all the previously raised concerns in their revised manuscript. I am happy to recommend the work for publication.
